# Global and regional burden of cancer in 2016 arising from occupational exposure to selected carcinogens: a systematic analysis for the Global Burden of Disease Study 2016

**DOI:** 10.1136/oemed-2019-106012

**Published:** 2020-02-13

**Authors:** GBD 2016 Occupational Carcinogens Collaborators, Tim Driscoll

**Keywords:** silica, asbestos, diesel engine exhaust, occupational exposure, epidemiology

## Abstract

**Objectives:**

This study provides a detailed analysis of the global and regional burden of cancer due to occupational carcinogens from the Global Burden of Disease 2016 study.

**Methods:**

The burden of cancer due to 14 International Agency for Research on Cancer Group 1 occupational carcinogens was estimated using the population attributable fraction, based on past population exposure prevalence and relative risks from the literature. The results were used to calculate attributable deaths and disability-adjusted life years (DALYs).

**Results:**

There were an estimated 349 000 (95% Uncertainty Interval 269 000 to 427 000) deaths and 7.2 (5.8 to 8.6) million DALYs in 2016 due to exposure to the included occupational carcinogens—3.9% (3.2% to 4.6%) of all cancer deaths and 3.4% (2.7% to 4.0%) of all cancer DALYs; 79% of deaths were of males and 88% were of people aged 55 –79 years. Lung cancer accounted for 86% of the deaths, mesothelioma for 7.9% and laryngeal cancer for 2.1%. Asbestos was responsible for the largest number of deaths due to occupational carcinogens (63%); other important risk factors were secondhand smoke (14%), silica (14%) and diesel engine exhaust (5%). The highest mortality rates were in high-income regions, largely due to asbestos-related cancers, whereas in other regions cancer deaths from secondhand smoke, silica and diesel engine exhaust were more prominent. From 1990 to 2016, there was a decrease in the rate for deaths (−10%) and DALYs (−15%) due to exposure to occupational carcinogens.

**Conclusions:**

Work-related carcinogens are responsible for considerable disease burden worldwide. The results provide guidance for prevention and control initiatives.

**Key messages**

**What is already known about this subject?**

- Occupational carcinogens have been shown to cause a considerable disease burden at national and global level. The last analysis of this issue at the global level was for the year 2000—this paper provides a new analysis for 2016.

**What are the new findings?**

- The study includes considerably more risk factor-outcome pairs compared to most previous burden of disease reports.
- The results highlight the important role of asbestos, diesel engine exhaust, second-hand smoke and silica in terms of occupational cancer burden.
- The burden of occupational cancer has increased considerably over the last two and a half decades, particularly due to ageing, changes in the proportion of workers exposed and population increases, with rates increasing for some exposures and not for others.

**How might this impact on policy or clinical practice in the foreseeable future?**

- Results of the present study highlight the need for urgent interventions to alleviate the global burden of occupational exposure to carcinogens, particularly asbestos.

## Introduction

Occupational carcinogens have been shown to cause a considerable disease burden at national and global level.^1–3^ The WHO Comparative Risk Assessment (CRA) project (2000) was the first attempt to produce comprehensive global estimates of the nature and extent of the burden of cancer arising from occupational exposures. In the year 2000, approximately 150 000 deaths were estimated due to past occupational exposure to 11carcinogens (three cancer outcomes—lung, mesothelioma and leukaemia).^1^

The series of Global Burden of Disease (GBD) studies conducted by the Institute of Health Metrics and Evaluation commenced with a focus on 2010. This GBD 2010 analysis^4^ included summary results for occupational carcinogenic risk factors,^4^ and the work has been updated several times at national and global level.^5–8^ The purpose of this paper is to describe in more detail the methods and results for the occupational carcinogens component of the GBD study, using the most recent comprehensive analysis, which was for 2016. This analysis, which included 14 occupational carcinogens and eight resulting cancers, covers many risk factor-cancer pairs that were not included in such global estimates prior to the GBD 2010 analysis. Accompanying papers provide an overview of all occupational risk factors included in the study^9^ and detailed information about chronic respiratory disease arising from non-infectious occupational airborne exposures^10^

## Methods

### General GBD methodology

The general methodology used in GBD 2016 is described elsewhere,^7 11 12^, as is the overall approach to occupational risk factors.^9^ These methods are briefly summarised here, and more detailed information is provided about the occupational carcinogen analysis.

The burden of occupational disease for each carcinogen-outcome pair was estimated using the population attributable fraction (PAF), that is, the proportion of deaths or disability-adjusted life years (DALYs) that would not have occurred if exposure was at the theoretical minimum risk exposure level (TMREL); this was then used to estimate attributable numbers of deaths or DALYs. The PAF requires information on the relative risk of the disease due to the exposure of interest and the proportion of the target population exposed. Per capita rates (directly standardised by age and sex) were based on persons aged 15 years and above. Results were calculated for all years from 1990 to 2016, inclusive; the 2016 findings are the focus of this paper. The sociodemographic index (SDI) is a composite indicator of development status based on total fertility rate, mean education for those aged 15 years and older and lag distributed income per capita.^7^ Region-specific, SDI-specific and global results are reported here. Country-specific information is available through the GBD Compare data visualisation.^13^ Employment data came from the International Labour Organization (ILO) Labour Force,^14^ supplemented where necessary by subnational data sources and modelling.

### Inclusion criteria

We included all International Agency for Research on Cancer (IARC) Group 1 (‘*carcinogenic to humans*’) carcinogens with relevant occupational exposure circumstances (as at 2014); a non-trivial number of cases, exposure level and proportion of persons exposed and available exposure data; and all associated cancer sites for these agents for which there was sufficient epidemiological evidence of a causation link (based on IARC’s assessments).

Exposure to 14 workplace carcinogens was included and linked to 8 cancer primary sites—breast (secondhand smoke (SHS: from tobacco smoking)), kidney (trichloroethylene), tracheal, bronchus and lung (‘lung’) (arsenic, asbestos, beryllium, cadmium, chromium VI, diesel engine exhaust, SHS, nickel, polycyclic aromatic hydrocarbons (PAHs), silica), larynx (asbestos, strong inorganic-acid mists), leukaemia (benzene, formaldehyde), mesothelioma (asbestos), nasopharynx (formaldehyde) and ovary (asbestos). With the exception of SHS and breast cancer (included as a pair in all GBD SHS burden estimates), selection of exposure-cancer pairs for inclusion was based on information in IARC Monographs 1–106^15^.

### Exposure

The exposure information was based primarily on the CAREX (Carcinogen Exposure) database, which provided a point estimate of industry-specific total prevalence of exposure to various carcinogens in countries of Western Europe from 1990 to 1993.^16^ We have assumed these circumstances not to have changed over the time period considered here. CAREX does not provide separate estimates by sex, age or non-Western European countries; thus, for a given industry, the same proportions were used across all these factors (online supplementary table S1). These proportions were distributed between ‘high’ and ‘low’ exposure based on information about exposure prevalence in high-income countries (countries in the Australasia, high-income North America, Western Europe and high-income Asia Pacific regions) and low-income and middle-income (LMI) countries (all other countries) from identified relevant cohort studies. On the basis of this information, the high to low CAREX exposure prevalence ratio was assumed to be 10:90 in high-income countries and 50:50 in LMI countries. This is considered in more detail in the online supplementary material.

10.1136/oemed-2019-106012.supp1 Supplementary data

To estimate age-specific numbers ever exposed during the risk exposure period, allowance was made for latency of the cancers and for workers who were no longer employed in an industry to still be at risk. To accomplish this, occupational turnover estimates (OTs) based on a risk exposure period defined by cancer latency (10–50 years for solid tumours (1966–2006), 0–20 years for haematopoietic cancers (1996–2016)), annual worker turnover estimates and normal life expectancy were developed and applied to the original prevalence data.^17^ Separate estimates are provided for men and for women, for the solid tumours (long latency) and haematopoietic (short latency) cancers, for 2016. Separate life tables (based on a representative country in each region) were used to estimate the OTs by region. The age assumptions and regional life expectancies determined the age distribution of the final exposed population. This is described in more detail in online supplementary material, appendices 1 and 2.

#### Asbestos exposure

To estimate the proportion ever exposed to asbestos, an asbestos impact ratio (AIR) approach (analogous to the smoking impact ratio approach described elsewhere^18^ was used in which rates of malignant mesothelioma were employed as a marker of asbestos exposure.

The AIR is defined as the excess deaths due to mesothelioma observed in that population divided by the excess deaths in a hypothetical population that is heavily exposed to asbestos and gives a measurement of the exposure level of a population to asbestos. We then used the AIR (as the estimate of exposure prevalence) and relative risks to calculate the PAF for each cause related to asbestos. Formally, the AIR is defined as:

$$AIR=\frac{C_{LC}-N_{LC}}{C_{LC}^{*}-N_{LC}}$$

where, for each country-sex group:

$C_{LC}$= mesothelioma mortality rate in the study population.

$N_{LC}$= mesothelioma mortality rate in a population not exposed to asbestos

$C_{LC}^{*}$= mesothelioma mortality rate in a population highly exposed to asbestos

Mortality rates for mesothelioma, *C*_*LC*_, by country, age and sex, were generated by causes of death models for GBD 2016.^11^ The background mortality of mesothelioma, *N*_*LC*_, was estimated using the model by Lin *et al*,^19^ which modelled mesothelioma rate against asbestos consumption. Using the uncertainty around the coefficients, we created 1000 draws of the mortality due to mesothelioma if there was no asbestos consumption in a country. The mean value for background mortality is 0.73 and 0.47 deaths per million males and females, respectively. We obtained the mortality rate for highly exposed individuals from asbestos workers, C*_LC_, from the meta-analysis by Goodman and colleagues.^20^ We used all studies in the meta-analysis that reported both the number of person-years followed and the number of cases of mesothelioma and found the death rate of all individuals included in the studies. The mesothelioma death rate for highly exposed individuals was estimated as 226 per million people. The AIR was used to calculate the exposure prevalence used for estimates of lung, ovarian and larynx cancer due to occupational exposure to asbestos. Custom PAFs were calculated for occupational causes of mesothelioma in the population of interest by using the ratio of excess mesothelioma mortality (*C*_*LC*_ *-N*_*L*__*C*_) in that population compared with the overall mesothelioma mortality rate (*C*_*LC*_) in that population.

### Relative risks

The relative risk estimates were primarily obtained from published meta-analyses or pooled studies or, where these did not exist, key single studies were used. Where single studies were used, the chosen study was the best-quality study with exposure circumstances that were assessed as most closely matching those assumed in the GBD study. The relative risks used in the analysis were chosen as much as possible to match an average ‘high’ exposure circumstance and ‘low’ exposure circumstance, assuming similarity of durations and intensities of exposure between the source data populations and world/national populations. For most exposures, appropriate low-level relative risks were not identifiable from the literature and in these cases were set to one. For all but one exposure-outcome pair, the same relative risk estimates were used for males and females and for all age groups (online supplementary table S2). For lung cancer arising from exposure to asbestos, separate relative risks were calculated for males and females based on estimates of cumulative exposure (as described in the online supplementary material). For non-asbestos exposures, relative risks (RRs) were set to 1.0 for ages 80 and over. The TMREL for each carcinogen-specific analysis was no exposure above background.

### Population attributable fraction

PAFs for all carcinogens except asbestos were estimated for each age-sex-country group using the equation based on Levin:^21^

$$PAF=\frac{\sum_{x=1}^{n}RR\left(x\right)P\left(x\right)-1}{\sum_{x=1}^{n}RR\left(x\right)P\left(x\right)}$$

where *P(x*) is the proportion of persons exposed at level *x* in the relevant population and *RR*(*x*) is the relative risk corresponding to exposure level *x*. For asbestos-related cancer, the above formula was used, substituting AIR for *P(x*).

Unless otherwise indicated, the PAFs presented in this paper are based on deaths.

### Modelling and calculation of uncertainty

The overall methodological approach and modelling used in the analyses, and the calculation and use of 95% uncertainty intervals (95% UI), were as described elsewhere.^7 9^ UIs are primarily presented in detail in the tables to assist with the flow of the text.

## Results

### Deaths

There were estimated to be 349 000 (95% UI 282 000 to 414 000) cancer deaths (3.9% of all cancer deaths; 79% male) in 2016 attributable to exposure to the occupational carcinogens evaluated. The deaths occurred primarily at older ages, with 88% occurring in people aged 55 years or older. Males had four times the rates of death compared with females, and the rates increased markedly with increasing age (online supplementary figure S1A).

The risk factors responsible for the highest proportion of deaths were asbestos (219 000 deaths; 62.7%), SHS (49 200; 14.1%), silica (48 000; 13.8%) and diesel engine exhaust (17 500; 5.0%) (table 1).

**Table 1:** Global occupation-attributable cancer deaths and DALYs by carcinogen and cancer type, 2016—number and per cent

Carcinogen	Deaths*	% of deaths	DALYs	% of DALYs
Arsenic†	8073 (2053–14 628)	2.3 (0.6–4.2)	219 218 (57 757–395 480)	3.0 (0.8–5.5)
Asbestos	218 827 (165 455–274 682)	62.7 (47.4–78.8)	3 556 876 (2 657 069–4 514 222)	49.4 (36.9–62.7)
Larynx cancer	3743 (2024*–*5528)		65 506 (35 042*–*99 124)	
Lung cancer	181 450 (128 287*–*236 621)		2 844 282 (1 957 872*–*3 803 219)	
Ovary cancer	6022 (2984*–*9404)		93 120 (45 796*–*149 948)	
Mesothelioma	27 612 (25 559*–*29 341)		553 967 (507 287*–*597 783)	
Benzene‡	1899 (596–3123)	0.5 (0.2–0.9)	83 867 (25 512–138 493)	1.2 (0.4–1.9)
Beryllium†	259 (213–312)	0.1 (0.1–0.1)	7223 (5886–8594)	0.1 (0.1–0.1)
Cadmium†	605 (504–709)	0.2 (0.1–0.2)	16 832 (14 142–19 639)	0.2 (0.2–0.3)
Chromium†	1276 (1126–1443)	0.4 (0.3–0.4)	35 452 (31 397–40 172)	0.5 (0.4–0.6)
Diesel engine exhaust†	17 500 (15 195–20 057)	5.0 (4.4–5.8)	485 693 (426 181–553 926)	6.7 (5.9–7.7)
Formaldehyde	1086 (900–1324)	0.3 (0.3–0.4)	46 932 (38 805–56 986)	0.7 (0.5–0.8)
Leukaemia	*608* (505*–*722)		27 914 (22 861*–*33 605)	
Nasopharynx cancer	*478* (330*–*685)		19 018 (12 994*–*27 091)	
Nickel†	8101 (1243–20 812)	2.3 (0.4–6.0)	221 352 (34 934–563 339)	3.1 (0.5–7.8)
Polycyclic aromatic hydrocarbons†	4526 (3826–5291)	1.3 (1.1–1.5)	125 779 (105 369–145 866)	1.7 (1.5–2.0)
Secondhand smoke	49 246 (25 336–80 957)	14.1 (7.3–22.2)	1 345 915 (703 984–2 186 305)	18.7 (9.8–30.4)
Breast cancer	4864 (1195–8401)		160 494 (39 883–276 832)	
Lung cancer	44 382 (20 655–75 463)		1 185 422 (551 749–2 013 661)	
Silica†	47 999 (21 235–75 452)	13.8 (6.1–21.6)	1 303 949 (576 291–2 042 004)	18.1 (8.0–28.4)
Strong inorganic-acid mists§	3535 (1520–6491)	1.0 (0.4–1.9)	105 226 (45 836–192 418)	1.5 (0.6–2.7)
Trichloroethylene¶	58 (13 –108)	0.0 (0.0–0.0)	1722 (379–3228)	0.0 (0.0–0.0)
Total**	348 741 (269 406–427 386)	100.0	7 199 850 (5 813 091–8 641 244)	100.0

*The numbers in brackets are 95% uncertainty intervals.

†Causes lung cancer.

‡Causes leukaemia.

§Causes laryngeal cancer.

¶ Causes kidney cancer.

**Numbers percentages add to more than 100 due to overlapping causes.

DALY, disability-adjusted life year.

The most common cancer primary sites were lung (300 000; 86.0%; due mainly to asbestos, diesel engine exhaust, silica, SHS, nickel and arsenic), mesothelioma (27 600; 7.9%; due to asbestos) and larynx (7200; 2.1%; due to asbestos and strong inorganic-acid mists) (table 2).

**Table 2:** Global occupation-attributable cancer deaths, DALYs and PAFs by cancer type and carcinogen, 2016—number and per cent

Cancer type	Deaths*			DALYs		
	N	% of deaths	PAF	N	% of DALYs	PAF
Breast cancer†	4864 (1195–8401)	1.4 (0.3–2.4)	0.9 (0.2–1.6)	160 494 (39 883–276 832)	2.2 (0.6–3.8)	1.1 (0.3–1.9))
Kidney cancer‡	58 (13*–*108)	0.0 (0.0–0.0)	0.0 (0.0–0.1)	1722 (379–3228)	0.0 (0.0–0.0)	0.1 (0–0.1)
Larynx cancer	7213 (4437–10 462)	2.1 (1.3–3.0)	6.5 (4.1–9.5)	169 127 (100 947–257 618)	2.3 (1.4–3.6)	6.2 (3.7–9.4)
Asbestos	3743			65 507		
Strong inorganic-acid mists	3535			105 226		
Leukaemia	2495 (1181–3734)	0.7 (0.3–1.1)	0.8 (0.4–1.2)	111 195 (52 577–166 086)	1.5 (0.7–2.3)	1.1 (0.5–1.6)
Benzene	1899			83 867		
Formaldehyde	*608*			27 914		
Lung cancer	299 998 (233 708–365 251)	86.0 (67.0–100.0)	17.6 (13.8–21.3)	6 091 207 (4 777 678–7 493 601)	84.6 (66.4–100.0)	16.7 (13.1–20.5)
Arsenic	8073			219 218		
Asbestos	181 450			2 844 282		
Beryllium	259			7223		
Cadmium	605			16 832		
Chromium	1276			35 452		
Diesel engine exhaust	17 500			485 693		
Nickel	8101			221 352		
Polycyclic aromatic hydrocarbons	4526			125 779		
Secondhand smoke	44 382			1 185 421		
Silica	47 999			1 303 949		
Mesothelioma§	27 612 (25 559*–*29 341)	7.9 (7.3–8.4)	91.4 (89.2–93.2)	553 967 (507 287–597 783)	7.7 (7.0–8.3)	83.8 (80.3–86.9)
Nasopharynx cancer¶	448 (330*–*685)	0.1 (0.1–0.2)	0.8 (0.5–1.1)	19 018 (12 994–27 091)	0.3 (0.2–0.4)	1.0 (0.7–1.4)
Ovary cancer§	6022 (2984–9404)	1.7 (0.9–2.7)	3.7 (1.8–5.7)	93 120 (45 796–149 948)	1.3 (0.6–2.1)	2.2 (1.0–3.5)
Total	348 741 (269 406–427 386)	100.0	3.9 (3.2–4.6)	7 199 850 (5 813 091–8 641 244)	100.0	3.4 (2.7–4.0)

*The numbers in brackets are 95% uncertainty intervals.

†Caused by second-hand smoke.

‡Caused by trichloroethylene.

§Caused by asbestos.

¶Caused by formaldehyde.

DALY, disability-adjusted life year; PAF, population-attributable fraction.

**Figure 1:**
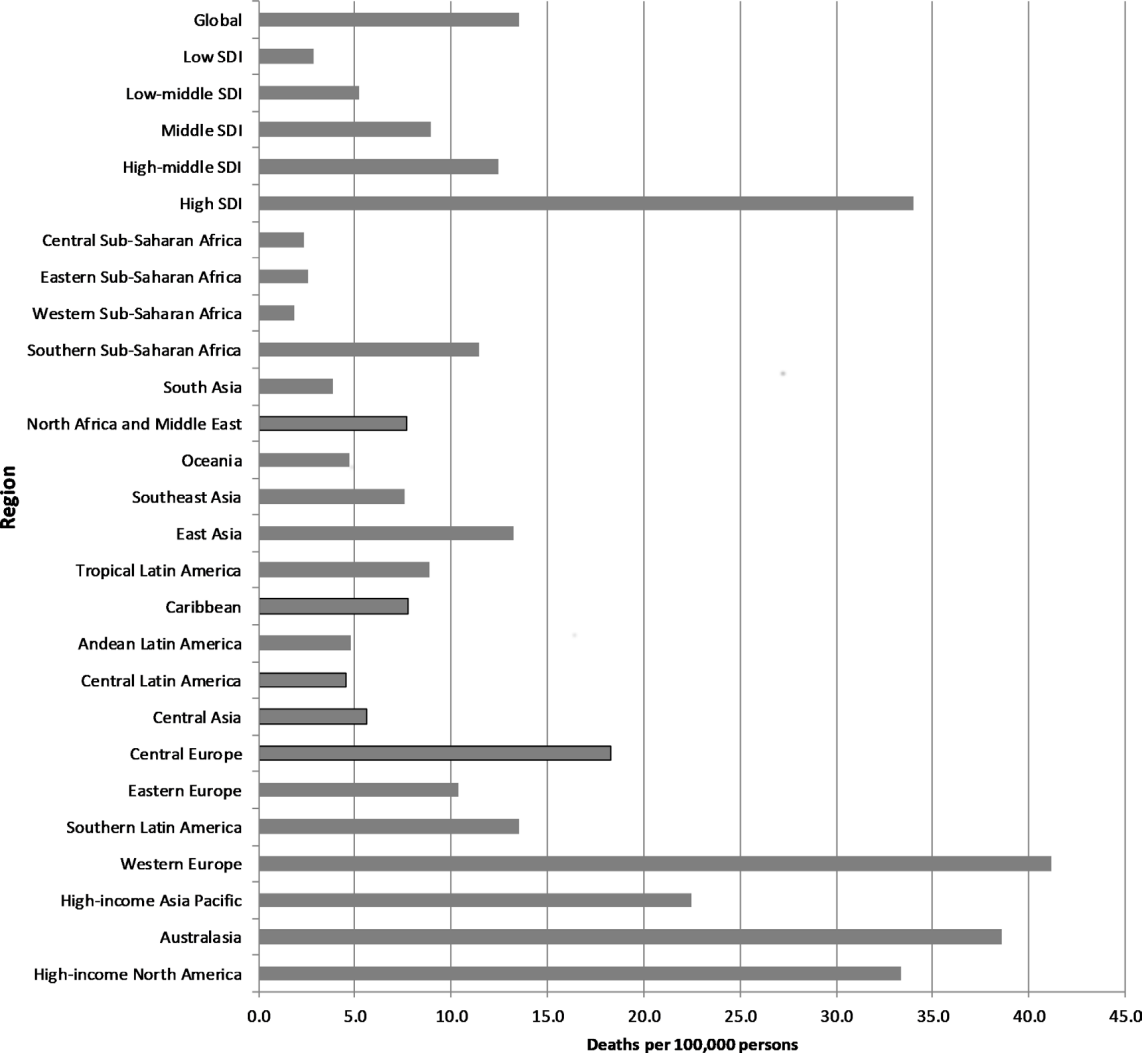
Occupation-attributable cancer deaths by region, 2016 (per 100 000 persons). Age-standardised; SDI=sociodemographic index.

The greatest number of deaths occurred in the Western Europe (92 400; 26.5%), East Asia (80 300; 23.0%) and high-income North America (56 200; 16.1%) regions. The highest per capita rates of death were in Western Europe, Australasia, high-income North America and high-income Asia Pacific (essentially the high SDI regions and largely due to asbestos-related cancers), and the lowest rates were in Western, Central and Eastern sub-Saharan Africa(part of the low-SDI quintile) (figure 1).

### DALYs

There were about 7.2 (95% UI 5.8 to 8.6) million DALYs in 2016 from exposure to occupational carcinogens, with the DALYs primarily driven by Years of Life Lost (due to premature deaths). The results for DALYs were qualitatively similar to those for deaths–77% occurring from male illness, rates being much higher in males and the rate increasing in older persons (although for DALYs the peak was at a slightly younger age than was the case for deaths) (online supplementary figure S1B); asbestos, SHS and silica being most commonly the causative risk factor (table 1); lung cancer and mesothelioma being the cancers most commonly caused (table 2) and East Asia and Western Europe being the regions with the largest number of DALYs. The regions with the highest rates were essentially the high SDI regions—Western Europe, Australasia and high-income North America and the lowest rates were again in Western, Eastern and Central sub-Saharan Africa (online supplementary figure S2).

### Asbestos

**Figure 2:**
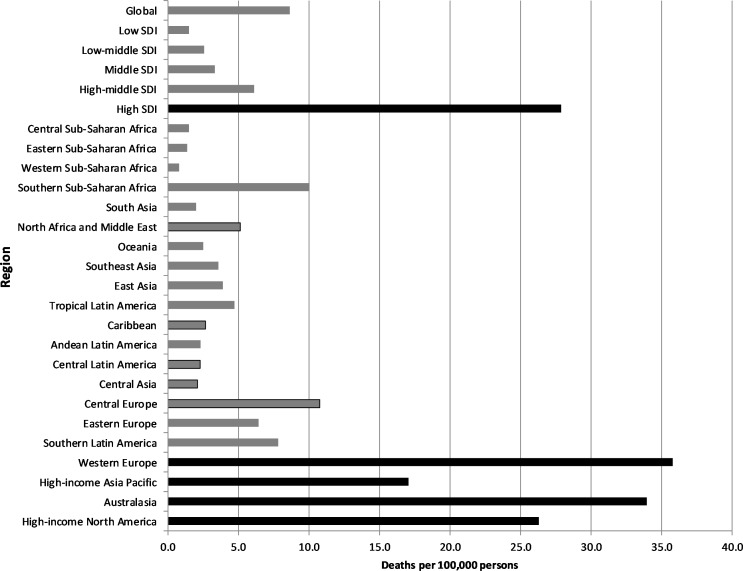
Occupational asbestos-related cancer deaths by region, 2016 (per 100 000 persons). The black bars highlight the high-income regions. Age-standardised; SDI=sociodemographic index.

Asbestos was the predominant carcinogen in terms of burden. The different patterns of use of asbestos are clearly reflected in the dominance of asbestos-related cancers in high-income regions, where asbestos use peaked three to four decades ago, in contrast to many of the LMI regions, where use became more common recently and is continuing.^22 23^ Asbestos-related cancers were responsible for 78%–88% of all occupational cancer deaths in the four high-income regions and 86% in Southern sub-Saharan Africa, compared to an average for all other regions of 48% (online supplementary table S3). These differences were even more evident when deaths were examined on a per capita basis, with high-income countries having by far the highest rates of asbestos-related cancer and the rates in Australasia and Western Europe being about 10 times the average rate in the remaining regions (figure 2). In the LMI countries, deaths from SHS, silica and diesel engine exhaust were consequently more prominent than asbestos-related deaths. A similar pattern was seen with DALYs.

### Population attributable fractions

The overall PAF for occupational carcinogens was 3.9% for deaths (3.4%for DALYs). This was higher for males (5.3%) than females (2.0%). The PAF increased with age up to age 75–79 years. The highest PAFs were for mesothelioma (91%), lung cancer (18%) and laryngeal cancer (6.5%) (table 2). The overall PAF varied considerably between regions, from lows of 0.7% in Western sub-Saharan Africa and 0.8% in Eastern sub-Saharan Africa, to highs of 8.9% in Australasia and 8.0% in Western Europe.

### Changes over time

There were 57% more deaths and 46% more DALYs due to occupational carcinogens in 2016 compared to 1990. There was a decrease in the rate for deaths (−10%) and DALYs (−15%). The changes varied widely across regions, with the rate of death in some regions (high-income Asia Pacific, South Asia and East Asia) increasing by 40%–60% over 26 years, and the rate in other regions (Eastern Europe, Central Asia and Andean Latin America) falling by 30%–40% over the same period (online supplementary table S4).

There was an increase over time in attributable deaths and DALYs arising from nearly all carcinogenic risk factors. The relevant rates increased for some risk factors (about 30% for chromium, diesel engine exhaust and PAHs), decreased for some (18% for strong inorganic-acid mists and 14% for asbestos) and showed little change for others. In terms of individual cancer primary sites(excluding kidney cancer due to very low numbers), the increase in deaths ranged from 33% for laryngeal cancer to 82% for mesothelioma. The rates for individual cancers decreased moderately (24% for laryngeal cancer) or showed little change (table 3).

**Table 3:** Change in global occupation-attributable deaths due to carcinogens, 1990 and 2016, number and rate (per 100 000 persons), by carcinogen and cancer type

Carcinogen	Deaths*			Deaths per 100 000 persons		
	1990	2016	% change	1990	2016	% change
Arsenic	4829 (883–9261)	8073 (2053–14 628)	67	0.3 (0.1– 0.6)	0.3 (0.1–0.5)	−2
Asbestos	145 235 (105 965–186 352	218 827 (165 455–274 682)	51	10.1 (7.4–12.9)	8.7 (6.6–10.9)	−14
Benzene	1177 (394–1920)	1899 (596–3123)	61	0.1 (0.0–0.1)	0.1 (0.0–0.1)	3
Beryllium	125 (102–150)	259 (213–312)	107	0.0 (0.0–0.0)	0.0 (0.0–0.0)	22
Cadmium	284 (237–333)	605 (504–709)	133	0.0 (0.0–0.0)	0.0 (0.0–0.0)	25
Chromium	578 (508–646)	1276 (1126–1443)	121	0.0 (0.0–0.0)	0.0 (0.0 0.1)	30
Diesel engine exhaust	7981 (6981–9119)	17 500 (15 195–20 057)	119	0.5 (0.4–0.6)	0.7 (0.6–0.7)	29
Formaldehyde	678 (562–818)	1086 (900–1324)	60	0.0 (0.0–0.0)	0.0 (0.0–0.0)	1
Nickel	4946 (563–13 968)	8101 (1243–20 812)	64	0.3 (0.0–0.9)	0.3 (0.0–0.8)	−4
Polycyclic aromatic hydrocarbons	2067 (1737–2421)	4526 (3826–5291)	119	0.1 (0.1–0.2)	0.2 (0.1–0.2)	29
Secondhand smoke	30 513 (15 914–49 666)	49 246 (25 336–80 957)	61	2.0 (1.1–3.3)	1.8 (0.9–3.0)	−9
Silica	30 680 (12 489–49 367)	47 999 (21 235–75 452)	56	1.9 (0.8–3.1)	1.8 (0.8–2.8)	−8
Strong inorganic-acid mists	2518 (1060–4645)	3535 (1520–6491)	40	0.2 (0.1–0.3)	0.1 (0.1–0.2)	−18
Trichloroethylene ­	21 (5–40)	58 (13–108)	169	0.0 (0.0 0.0)	0.0 (0.0 0.0)	58
Breast cancer	2695 (628–4698)	4864 (1195–8401)	81	0.2 (0.0–0.3)	0.2 (0.0–0.3)	2
Kidney cancer	21 (5–40)	58 (13–108)	169	0.0 (0.0–0.0)	0.0 (0.0–0.0)	58
Larynx cancer	5418 (3362–7918)	7213 (4437–10 462)	33	0.4 (0.2–0.5)	0.3 (0.2–0.4)	−24
Leukaemia	1551 (775–2300)	2495 (1181–3734)	61	0.1 (0.0–0.1)	0.1 (0.0–0.1)	3
Lung cancer	193 015 (150 197–237 598)	299 998 (233 708–365 251)	55	13.0 (10.2–16.0)	11.6 (9.1–14.2)	−11
Mesothelioma	15 206 (13 791–17 246)	27 612 (25 559–29 341)	82	1.0 (0.9–1.2)	1.1 (1.0–1.1)	4
Nasopharynx cancer	298 (209–410)	478 (330–685)	60	0.0 (0.0–0.0)	0.0 (0.0–0.0)	−2
Ovarian cancer	3845 (1905–6040)	6022 (2984–9404)	57	0.5 (0.2–0.7)	0.4 (0.2–0.7)	−10
All	222 049 (178 784–268 582)	348 741 (282 253–414 071)	57	15.0 (12.1–18.1)	13.5 (11.0–16.0)	−10

*The numbers in brackets are 95% uncertainty intervals.

## Discussion

This analysis has shown that occupational exposure to carcinogens is an important cause of death and disability across the world. There were an estimated 349 000 deaths and 7.2 million DALYs in 2016 due to these exposures. All regions had considerable numbers of deaths and DALYs, but the relative burden varied across regions and ages and by sex. Key considerations regarding the study and its implications are presented here. These issues are considered in more detail in online supplementary appendix 3.

### Risk factors

The main risk factors responsible for the deaths were asbestos, SHS and silica, with lung cancer being the predominant outcome for each of these exposures. Overall, 14 different occupational carcinogens were included in the analysis. Recent work in several countries suggests many such exposures remain in high-income countries.^24–28^ Although there is limited information about exposures in LMI countries, it is reasonable to expect that such exposures there would commonly be less well controlled and probably more prevalent due to fewer automated facilities.^29^

The legacy of asbestos is clear from the analysis, with an estimated 219 000 deaths each year from asbestos-related cancer (note that this does not include deaths from asbestosis). In high-income countries, there has been considerable effort in the last three decades to minimise exposure to asbestos. Unfortunately, even if exposure to asbestos was to cease completely, deaths from asbestos-related cancers would be expected to continue for the next four to five decades. While asbestos control has improved greatly in high-income countries, there are still many instances of exposure,^24 26 27^ sometimes inadvertent and sometimes seemingly through poor occupational health and safety practices. Of even more concern is the continued use of asbestos, primarily in LMI countries, often with very poor exposure control.^30^ Regions such as South Asia and East Asia have used increasing amounts of asbestos in recent decades and are still using it in a variety of occupational circumstances.^23 30 31^ This, combined with their large workforces, means the identified deaths from asbestos-related cancers such as mesothelioma (a cancer with very long latency) in these regions are a forerunner of what can be expected to be a far higher number of deaths in the coming decades. Even in some high-income countries, mesothelioma incidence has not yet peaked (England^32^) or appears to have only recently peaked (Australia,^33^ Canada,^34^ Italy,^35^ Slovenia^36^).

### Comparison with other studies

Lower estimates in the CRA 2000 study^1^(which estimated half the number of cancer deaths) and higher absolute or equivalent estimates in other global or national studies^2 3 34^ arise from differences in methodologies, particularly in terms of the risk factors and outcomes included, the approach to estimating the population at risk, and the approach to estimating the prevalence of exposure to asbestos, all of which are considered to be improved in the current study compared with previous studies.

### Methodological considerations and limitations

Methodological issues relevant to the overall study are considered in detail in the occupational risk factors overview paper.^9^ The main aspects relevant to the carcinogen analysis included the exclusion of some relevant IARC Group 1 exposures, for example, UV exposure from sunlight (associated with skin cancers) and welding fumes (associated with lung cancer) as well as IARC Group 2A exposures (‘*probably carcinogenic to humans*’) and cancer sites with limited (as determined by IARC) epidemiological evidence of a causal connection to included exposures (the most important exclusions in terms of numbers of deaths are likely to be shift work, with breast cancer the associated outcome and occupational exposure to the ultraviolet component of sunlight, leading to skin cancer); probable under-recognition of occupational carcinogens;^37^ assumptions regarding latency, turnover and at-risk period; the reliance on the CAREX database for exposure prevalence estimates; the method used for estimation of relative risk for lung cancer from asbestos exposure; the potential for mismatch between the relative risk estimates used and the exposure circumstances to which they have been applied; the exclusion of people 80 years or older from the non-asbestos cancer estimates (which was due to an error in programming); suspected overall underestimation of mesothelioma occurrence but possible overestimation due to the assumption that all mesothelioma above background occurrence is a product of occupational asbestos exposure and not explicitly taking account of possible interactions between occupational and other risk factors in people exposed to multiple risk factors. Of the 47 occupational carcinogenic exposures identified in a recent article reviewing IARC Monograph classifications up to 2017,^37^ 14 were included in this analysis. The remainder were excluded because of one or more of being classified as Group 1 after 2014 (eg, welding); lack of suitable exposure data (eg, ionising radiation, which accounted for nine of the 47); probable insufficient number of cases (eg, benzidine) and insufficient exposure level and/or proportion of persons exposed (eg, Bis(chloromethyl)ether).

### Implications of the data

The results presented here serve to emphasise the importance of eliminating occupational exposure to asbestos, given the continuing legacy of past exposure in those countries that have banned use and the likelihood that countries still using it will face the same issues in future years. They also highlight the need for all countries and relevant international agencies to work to eliminate or control occupational exposure to carcinogens, which is inadequate in many LMI countries and sometimes of the order of the high exposures that were experienced in past decades in high income countries. Suitable approaches include adopting and enforcing relevant legislation; further development of global and regional frameworks for control of occupational carcinogens; strengthening exposure and outcome data collection and reporting at the country level and emphasising the importance of primary prevention.^38^

## Conclusion

Work-related carcinogens are responsible for considerable disease burden worldwide. Several exposures result in major burden, and the total burden has worsened in the last two decades, although it has decreased for some exposures on a per capita basis. The current burden largely reflects exposures from past decades, but there is sound evidence that many such exposures continue in current workplaces. The results provide guidance for prevention and control initiatives that are clearly needed.

10.1136/oemed-2019-106012.supp2 Supplementary data
