# Supplementary material for: Global and regional burden of cancer in 2016 arising from occupational exposure to selected carcinogens: a systematic analysis for the Global Burden of Disease Study 2016
Source: Occup Environ Med. 2020 Feb 13;77(3):151–9. doi: 10.1136/oemed-2019-106012 (PMC7035689; doi:10.1136/oemed-2019-106012)
Supplement: Supplementary data [file oemed-2019-106012supp001.pdf]

Global and regional burden of cancer in 2016 arising from occupational exposure to carcinogens: a systematic analysis for the Global Burden of Disease Study 2016

GBD 2016 Occupational Exposure to Carcinogens Collaborators.

SUPPLEMENTARY MATERIAL

. *Occup Environ Med* 2020; 77:151–159. doi: 10.1136/oemed-2019-106012

APPENDIX 1: DETAILED INFORMATION ON METHODOLOGY

Estimating the proportion of the population exposed

The data available for estimating the exposed population were, for each GBD region, population estimates by sex and age group (P_i for age group i); estimates of economically active proportions (pEA_i) by age and sex; estimates of the proportions employed in nine industry groups (pW_j for industry group j); and data from CAREX of the proportions of workers within these industry groups actually exposed to the carcinogenic agents of interest (p_wE_{jk} for carcinogenic agent k). These estimates were combined to produce a point (prevalence) estimate of numbers exposed by sex, industry group (j), and agent (k) in each region as:

$$n_{jk} = \Sigma_i(P_i * pEA_i) * pW_j * p_wE_{jk} \qquad (1)$$

It was also assumed that for high-income regions, proportions exposed at high and low levels were 0.1 and 0.9, respectively, and for LMI country regions the proportions were 0.5 and 0.5,based on information about exposure prevalence in high-income countries (countries in the Australasia, high-income North America, Western Europe, and high-income Asia Pacific regions) and low- and middle-income (LMI) countries (all other countries) from identified relevant cohort studies[1-7].

The turnover methodology used to estimate numbers ever exposed is based on the estimation method used in the CRA 2000 study[8]. For estimating population attributable fraction (PAF), the proportion of the population exposed to a workplace carcinogen is estimated as the numbers who have ever been exposed for at least a year during the risk exposure period (REP) and are still alive in the target year (say 2010), divided by the numbers who were of working age during that risk period and are still alive in that year. I.e., it is the proportion of the population at risk of developing this occupational cancer at the specific site in the target year. For these estimates, occupational turnover was estimated by age group, leading to separate age-specific PAF estimates that were applied to age-group-specific total cancer deaths (or DALYs).

The period during which exposure occurred that was relevant to the development of the cancer in the target year, the risk exposure period (REP), is defined by cancer latency, assumed to be 10-50 years for solid tumours (giving a REP for 2010 of 1961–2000, for example) and 0-20 years for haematopoietic neoplasms (giving a 2010 REP of 1991–2010)[9].

. *Occup Environ Med* 2020; 77:151–159. doi: 10.1136/oemed-2019-106012

Numbers ever exposed were estimated taking into account annual staff turnover, life expectancy, age at recruitment (15-44 assumed) and age at retirement (65 for men and women), using the "turnover equation" for age group a and agent k:

$$N_{e(REP)ak} = \sum_{i=a}^{i=b} [l_{(adj15)i} * n_k/(R-15)] \qquad + \sum_{k=0}^{k=(age(u)-age(1))} \sum_{j=c+k}^{j=d+k} [l_{(adj15)j} * n_k * OT/(age(u)-age(l)+1)] \qquad (2)$$

where n_k is the point estimate of numbers exposed to agent k from equation (1), OT is staff turnover per year, used for all regions in the absence of country-specific data, based on the Great Britain study[9,10] and defined as new starters continuing in employment for at least one year as a proportion of all employed for at least a year (0.1 for men, 0.14 for women). R is retirement age (65), $l_{(adj15)i}$ is the proportion of survivors up to age i from sex, country, and time-appropriate WHO Life Table data, adjusted to include only those still alive at age 15 by taking $l_{(adj15)i} = l_i / l_{15}$. Tables representing current survival for the target year and regions were used to enable results to be compared between regions. a to b is the age range achieved by the original cohort members, and c to d by the turnover recruited cohort members, by the target year, and age(u) and age(l) are the upper and lower recruitment age limits (15 to 44 used here).

In practice, an occupational turnover factor ($OT_a=N_{e(REP)ak}/n_k$) was estimated for each combination of period-, sex-, region- and age-specific parameters and applied to the point estimate of numbers exposed. These factors represent the ratio between a point estimate and ever-exposed estimate of exposed workers; see Table A1 for examples of the OT_a factors. The proportion of the population ever exposed in the REP to each carcinogenic agent was obtained, by age group and sex, as:

$p(E)_{ak} = N_{e(REP)ak}/N_{p(REP)a} = OT_a*n_k/N_{p(REP)a}$ for age group a

where $N_{p(REP)a}$ is the age-sex-group-specific estimates of the population ever of working age in the REP and alive in the target year.

Although $\Sigma_a OT_a *n_k = N_{e(REP)k}$ for all ages, the age-specific PAFs cannot be summed across age groups as the age-specific $p(E)_{ak}$s from which they are estimated do not have a common ($N_{p(REP)}$) denominator, although attributable numbers can be summed. Note that the age in the target year of those ever exposed during the REP is determined entirely by assumptions about the age of recruitment into the exposed workforce, retirement age, and regional life expectancies. Therefore, a total (all-age) point estimate of the economically active population is used to obtain the ever-

. *Occup Environ Med* 2020; 77:151–159. doi: 10.1136/oemed-2019-106012

exposed estimate, rather than taking account of the current age structure of the economically active population. For consistency, the same entry age assumptions have been used across all regions, with entry into the exposed workforce assumed to be distributed uniformly between the ages of 15 and 44. Using separate life expectancy estimates for countries representing each of the GBD regions takes some account of differences in age profiles of workers across the regions.

Table A1 Examples of occupational turnover (OT) factors by age(1)

region	Western Europe		Asia East		Eastern Europe		Sub-Saharan Africa, Southern	
country	Germany		China		Russia		South Africa	
year	2008	2008	2008	2008	2008	2008	2008	2008
sex	Male	Female	Male	Female	Male	Female	Male	Female
15 – 19	0.00	0.00	0.00	0.00	0.00	0.00	0.00	0.00
20 – 24	0.00	0.00	0.00	0.00	0.00	0.00	0.00	0.00
25 – 34	0.25	0.35	0.25	0.35	0.24	0.35	0.23	0.32
35 – 44	0.58	0.81	0.57	0.80	0.52	0.79	0.46	0.64
45 – 54	0.89	1.26	0.87	1.24	0.74	1.20	0.58	0.84
55 – 64	0.92	1.34	0.88	1.30	0.65	1.23	0.48	0.77
65 – 74	0.77	1.12	0.70	1.05	0.42	0.95	0.31	0.53
75 – 84	0.37	0.60	0.29	0.48	0.13	0.42	0.11	0.20
85+	0.09	0.16	0.04	0.08	0.02	0.07	0.02	0.03
All ages(2)	3.85	5.65	3.60	5.31	2.72	5.01	2.18	3.33

(1) The age-specific OT factors are a product of (1) the multiplier which gives the estimate of overall numbers based on REP length, staff turnover, and numbers missing by the target year due to exceeded life expectancy, which varies with entry cohort age assumptions, and (2) the proportion of $N_{e(REP)}$ falling in the specific age group by the target year, which is determined mainly by age at recruitment assumptions, plus a minor contribution from the entry cohort age profile assumption.
(2) Element (1), which is the "all age" factor, remains constant across age groups.

Age-sex-specific attributable fractions are then estimated (summed over industry groups j and exposure levels h) as

$PAF_{ak} = \Sigma_h(\Sigma_j p(E)_{akjh}*(RR_{kh}-1)) / [1+\Sigma_h(\Sigma_j p(E)_{akjh}*(RR_{kh}-1))]$ for age group a

where RR_{kh} is the relative risk for the specific cancer for agent k at exposure level h. The PAFs are applied to target year cancer numbers (deaths or DALYs) for that specific age group.

A combined PAF across agent exposures k, by sex and region, is estimated[11] as

$PAF_{combined,a} = 1-\Pi_k(1-PAF_{ak})$ for age group a

To use this equation, the assumption was made that where the exposures overlapped in the working population, the exposures were independent and their joint effect on initiating or promoting cancer was multiplicative, i.e., $RR_{(exp\ 1\ and\ exp2)} = RR_{(exp1)} * RR_{(exp2)}$. (Some bias is introduced where the assumptions are unmet, although this can be estimated. This is also an appropriate equation to use for combining disjoint exposure PAFs, as it introduces less bias into the results compared with, for example, direct summing of the PAFs (Hutchings – personal communication). To estimate overall (all-age) PAFs, for individual agents or combined across agents, the summed attributable numbers are divided by the cancer numbers summed across age groups.

Estimating cumulative exposure to asbestos

The relative risk of lung cancer resulting from asbestos exposure was based on an estimate of cumulative exposure to asbestos. The relevant cumulative exposures to use were estimated for this study by first estimating the duration of exposure for each age group and using this information to estimate the average cumulative exposure, assuming all were exposed at the relevant United States Permissible Exposure Limit (PEL) in operation at the relevant time.

To estimate average duration of exposure by age group, the age at which workers born from 1920 onward enter the exposed cohort was allocated at random between a minimum 15 and maximum age 44. The workers' age in the target year (2010), their maximum possible duration of exposure up to retirement, and a (lognormally distributed) random duration of exposure[1] applied to the time between recruitment and the target year or retirement were used to estimate the average duration of exposure in five-year age groups (using 90 (year) *1000 Monte Carlo runs). The results are shown in Table A2. Recruitment from the 1920 birth cohort has been used in this update of the 2000 CRA methodology to allow all ages (to 105) into the entry cohort to avoid cutting off long durations of exposure of up to 50 years for entry at 15 and retirement at 65. Along with a cutoff at age 95, this accommodated annual turnover recruitment in, for example, a 15-44 age range, without causing durations to be cut off prematurely at higher ages.

Then cumulative exposure, for example, to asbestos was the product of the number of years of work (average duration of exposure) and the average exposure level during that period. The average exposure level was estimated using the relevant United States Permissible Exposure Limit (PEL) to asbestos in operation during the relevant years and taking (a multiple of) the weighted average

[1] A number between zero and 1 obtained as the inverse minus 1 of a lognormal cumulative distribution function of x, where ln(x) is normally distributed with mean = ln(1.5) and standard deviation = (ln(2)-ln(1))/6.

. *Occup Environ Med* 2020; 77:151–159. doi: 10.1136/oemed-2019-106012

based on the age of the person in the target year and thus the PEL that was in operation during the years the person could have worked, in fibres/ml (f/ml). The results are in Table A2. Multiples of 2.0 (high) and 0.5 (low) for men and 1 (high), 0.2 (low) for women were used for all regions, based on an assessment taking into account relevant exposure data in published literature.

Table A2 Average duration and level of exposure to asbestos, by WHO age group

Age	Average duration of exposure (years)	mean f/ml at PEL, asbestos
15-19	1.0	0.10
20-24	2.1	0.10
25-34	4.2	0.11
35-44	6.6	0.28
45-54	10.5	0.94
55-64	15.5	2.57
65-74	18.1	4.78
75-84	18.2	7.13
85+	18.1	8.89
All ages	13.6	5.93

Relative risk for asbestos-related lung cancer

The majority of the relative risks were based on "exposed versus non-exposed" results. However, for lung cancer arising from exposure to asbestos, separate relative risks were calculated for high and low cumulative exposure levels (described in the Supplementary Matter). a relative risk for cumulative exposure at high level (assuming a United States Permissible Exposure Level (PEL) multiplied by two for men and one for women) and low level (PEL*0.5 for men, PEL*0.2 for women) was estimated from the study by Lenters and colleagues[12], based on a meta-analysis of the five most methodologically sound studies, using their formula:

$RR = \alpha(1 + K_L \times CE)$

where: α=1.44,

K_L= the slope of increase in the RR per unit of cumulative exposure (CE) to asbestos (in fibre-years per ml) = 0.0036, and

CE = (average level of exposure) * (average years of exposure) * PEL multiplier = 13.6 * 5.93 * PEL multiplier (see Table A2 in Appendix 1).

. *Occup Environ Med* 2020; 77:151–159. doi: 10.1136/oemed-2019-106012

APPENDIX 2: WORKED EXAMPLE

Introduction

This example provides a step-by-step description of the approach to estimating PAFs, deaths, and DALYs, using diesel engine exhaust (DEE) exposure in males aged 55-64 years in Eastern Europe as the focus. This is based on data from GBD 2010 rather than GBD 2016.

Risk factor – exposure evidence

Diesel engine exhaust is a Group 1 IARC carcinogen, with sufficient epidemiological evidence that DEE causes lung cancer in humans[13].

Exposure

The proportion of workers in each industry came from the ILO database[14] for the year for which the burden estimates were being made (2010 in the current example) (Table A3 – Column 2). The same estimates were used for all ages, but different estimates were available by sex. The proportion of persons within an industry who were exposed came from CAREX[15] (Table A3 – Column 3). The same estimates were used for all ages and both sexes.

The proportion of persons in the workforce who were exposed was estimated by multiplying the proportion of workers in each industry by the proportion of persons within an industry who were exposed (Column 2 * Column 3) (Table A3 – Column 4).

The number of persons in the current workforce aged 55-64 and estimated to have been exposed in 2010, stratified by the industry in which the persons worked was then calculated. This number was estimated by multiplying the proportion of persons in the workforce who were exposed (Column 4) by the total number of people in the workforce (for Eastern Europe in 2010, the number of male workers in the workforce was 48,740,000, which came from workforce data not shown in Table A3) (Table A3 – Column 5).

The total number of people ever exposed (and so at risk) by 2010 will be higher than the number currently exposed because people remain at risk after they are exposed. That is, people who have left the workforce remain at risk for some period of time (often decades) afterwards. Using estimates of workforce turnover and life expectancy (as described in Section A1.4), the number of people ever exposed over a period of 10-50 years prior to 2010 and still alive in 2010, as a proportion of the currently exposed workforce, was estimated, both overall and by age group.

Table A3 Summary of calculations to produce the PAF for DEE and lung cancer for males aged 55-64 years in Eastern Europe

1 Industry	2 Prop. in industry	3 Prop. currently exposed	4 Prop exp to DEE	5 Number currently exposed#	6 Number ever exposed#	7 Prop. ever exposed	8 Prop. with high exposure	9 Prop. with low exposure	10 Col 8 *(RR-1)	11 Col 9 *(RR-1)
1 Agriculture, hunting, forestry, and fishing	0.111	0.0066	0.00072	34.88	22.71	0.00300	0.00150	0.00150	0.00071	0
2 Mining and quarrying	0.026	0.2200	0.00573	279.16	181.79	0.02402	0.01201	0.01201	0.00564	0
3 Manufacturing	0.209	0.0119	0.00250	121.61	79.19	0.01046	0.00523	0.00523	0.00246	0
4 Electricity, gas, and water	0.039	0.0336	0.00132	64.08	41.73	0.00551	0.00276	0.00276	0.00130	0
5 Construction	0.096	0.0582	0.00560	272.96	177.75	0.02349	0.01174	0.01174	0.00552	0
6 Wholesale and retail trade and restaurants and hotels	0.140	0.0048	0.00068	33.09	21.55	0.00285	0.00142	0.00142	0.00067	0
7 Transport, storage, and communication	0.128	0.1343	0.01716	836.31	544.60	0.07196	0.03598	0.03598	0.01691	0
8 Financing, insurance, real estate, and business services	0.081	0.0000	0.00000	0.00	0.00	0.00000	0.00000	0.00000	0.00000	0
9 Community, social, and personal services	0.170	0.0092	0.00157	76.31	49.69	0.00657	0.00328	0.00328	0.00154	
Total	***1.000***								***0.03475***	***0***

#: '000s.

Column 2 = Proportion of workers who are currently (in 2010) employed in each industry sector (from ILO database).
Column 3 = Proportion of the workers in each industry who are currently (in 2010) exposed to DEE (from CAREX).
Column 4 = Proportion of the total workforce who are in each industry and who are currently (in 2010) exposed to DEE (= Column 2 * Column 3).
Column 5 = Number of workers currently (in 2010) exposed (= Column 4 * total number of people in the workforce) (from ILO database).
Column 6 = Number of persons ever (in the period 1960 to 2000) exposed through work and still alive in 2010: by industry (= Column 5 X age-specific turnover factor in Table A4).
Column 7 = Proportion of persons ever (in the period 1960 to 2000) exposed through work and still alive in 2010: by industry (=Column 6 / total number of people in the workforce in 2010).
Column 8 = Proportion of persons ever (in the period 1960 to 2000) exposed at high level through work and still alive in 2010: by industry (= Column 7 * 0.5).
Column 9 = Proportion of persons ever (in the period 1960 to 2000) exposed at low level through work and still alive in 2010: by industry (= Column 7 * 0.5).
Column 10 = Proportion of persons ever exposed (in the period 1960 to 2000) at high level through work and still alive in 2010 multiplied by ((RR for high exposures) – 1): by industry.
Column 11 = Proportion of persons ever exposed (in the period 1960 to 2000) at low level through work and still alive in 2010 multiplied by ((RR for low exposures) – 1): by industry.

For Eastern Europe, this overall proportion was 2.72 (Table A4). That means that the total number of persons ever exposed and still alive in Eastern Europe in 2010 was estimated to be 2.72 times the number of persons in the workforce who were currently exposed to diesel engine exhaust in 2010. These ever-exposed persons were of various ages. The number of persons in a given age group now (i.e., 2010, the year we are estimating the burden for) who had ever been exposed and who were still alive was estimated by multiplying the number of persons currently exposed (Column 5) by the relevant turnover proportion for that age group (which for the 55-64 year age group was 0.65 – Table A3) (Table A3, Column 6).

The proportion of the population who had ever been exposed, by age group, was estimated by dividing the number ever exposed in a given age group (Table A3 - Column 6) by the total number of persons in that age group (which came from population data, not shown in Table A3) (Table A3, Column 7).

The proportion ever exposed was divided into high-exposed and low-exposed on the basis of 50:50 (high:low) in LMI countries and 10:90 (high:low) in high-income countries. For this analysis, Eastern Europe was considered to be a LMI country region, so the proportions with high exposure were estimated by multiplying Column 7 in Table A3 by 0.5 (Table A3, Column 8), and the proportions with low exposure were also estimated by multiplying Column 7 by 0.5 (Table A3 – Column 9).

Table A4 Turnover factors for Eastern Europe, by age

**Age group**	**Turnover**
15 – 19 years	0.00
20 – 24 years	0.00
25 – 34 years	0.24
35 – 44 years	0.52
45 – 54 years	0.74
55 – 64 years	0.65
65 – 74 years	0.42
75 – 84 years	0.13
85+ years	0.02
All ages	***2.72***

. *Occup Environ Med* 2020; 77:151–159. doi: 10.1136/oemed-2019-106012

Risk estimate

The high exposure RR for lung cancer arising from DEE was 1.47 (85% CI 1.29–1.67). This estimate came from a meta-analysis by Lipsett and Campleman, 1999[16]. The low exposure RR was set to 1.0 (i.e., equal to background risk).

Calculating age-specific PAFs

To estimate the PAF (Exposed*(RR-1)/[Exposed*(RR-1)+1]), the quantity [Exposed*(RR minus one)] was calculated. To do this, the proportion ever exposed, by age group, was multiplied by (RR minus one) for each industry and summed. For high exposure, the RR was 1.47. So, the proportion ever exposed (Table A3, Column 8) was multiplied by 0.47 (Table A3, Column 10). The same occurred for low exposure. However, the low exposure RR was 1.0 (this was true for diesel, but not for all exposures), which meant the proportion ever exposed at low levels was multiplied by zero (Table A3, Column 11).

The final PAR for the 55- to 64-year-old age group in males in Eastern Europe was estimated by adding the total of columns 10 and 11 and inserting these in the PAF formula:
Sum[(Exposed*(RR-1)] / [1 + Sum(Exposed*(RR-1)].

In this instance, the values were 0.03475/(1 + 0.03475) = 0.034 = PAF (for age 55-64).

Calculating deaths, DALYs, and overall PAF

The age-specific PAF (Table A5, Column 3) was multiplied by the number of deaths from lung cancer estimated by the GBD 2010 project for males aged 55-64 years in Eastern Europe (Table A5, Column 2). This provided the estimate of the number of deaths from lung cancer due to DEE in males aged 55-64 years in Eastern Europe (757: Table A5, Column 4). This approach was repeated for all age groups.

The age-specific attributable lung cancer deaths for males were added, to produce the total number of deaths from lung cancer due to DEE in males in Eastern Europe (1,919: Table A5, Column 4, bottom row). This total was divided by the total number of deaths from lung cancer in Eastern Europe (658,977: Table A5, Column 2, bottom row) to produce the all-age PAF for DEE and lung cancer in Eastern Europe (2.9%: Table A5, Column 3, bottom row). The same approach was used for both sexes and all regions to estimate the relevant number of deaths and the PAFs by age and sex and overall.

DALYs were calculated by multiplying the relevant age-sex-specific PAF by the number of DALYs from lung cancer estimated by the GBD 2010 project for the relevant age and sex groups in the region (calculations not shown).

. *Occup Environ Med* 2020; 77:151–159. doi: 10.1136/oemed-2019-106012

Table A5 **Summary of calculations to produce the overall PAF for DEE and lung cancer for males in Eastern Europe.**

1<br>Age	2<br>Deaths (all)	3<br>PAF (%)	4<br>Deaths (occ)
15-24	36	0	0.0
25-34	183	0.7	1.3
35-44	1173	1.6	18.6
45-54	10,350	2.1	220.9
55-64	22,541	3.4	757.0
65-74	20,927	3.2	662.1
75+	10,688	2.4	260.2
Total	***658,977***	***2.9***	***1,919***

Column 2 = Deaths from lung cancer from all causes (estimated by GBD 2010).

Column 3 = Population attributable fraction for lung cancer due to DEE (estimated by the current project).

Column 4 = Deaths from lung cancer due to exposure to DEE (= Column 2 * Column 3).

The shaded box shows the overall PAF, calculated by dividing the bottom cell in Column 4 by the bottom cell in Column 2.

. *Occup Environ Med* 2020; 77:151–159. doi: 10.1136/oemed-2019-106012

APPENDIX 3: FURTHER DISCUSSION

Risk factors

Exposure to second-hand smoke is now well-recognised as an important risk factor for lung cancer and for several decades has been the focus of efforts to decrease exposure[17-19]. As for asbestos, the results from this study primarily reflect the result of exposures before many of the current control measures were implemented. They also serve to show the effect of not having appropriate control measures in place, as is commonly still the case in many LMI countries[20] and for some occupations in high-income countries[17,18].

Silica is a common exposure in a range of occupational situations[10,21,22]. There has been extensive debate as to its human carcinogenicity[6,23], but silica appears to be responsible for a considerable burden due to occupational exposures.

Diesel engine exhaust has many similarities to silica. Like silica, it is a common exposure in the occupational environment and its potential harm has probably not been fully appreciated[24], diesel engine exhaust having only been formally recognized as a definite human carcinogen in 2012[13]. Although the relative risk of lung cancer associated with diesel engine exhaust is not high, the high prevalence of past and current exposure means the cancer burden resulting from it is significant.

Our primary analysis did not include any IARC Group 2A exposures ("*probably carcinogenic to humans*"), or cancer sites with limited (as determined by IARC) epidemiological evidence of a causal connection to included exposures. This decision was based on an assessment by the GBD central team that there was insufficient evidence to allow other exposures and outcomes to be included, given the evidence requirements for exposures and outcomes in the overall GBD study. However, an additional analysis using similar methods was able to be undertaken to provide an indication of the potential contribution from these additional exposures and outcomes. This identified an additional 97,000 deaths (about 28% more than the official GBD estimate), in particular resulting from second-hand smoke (due to cancer of the larynx and pharynx), asbestos (cancer of the stomach and colon), and lead (cancer of the stomach and lung).

Based on these results, the highest PAFs apart from mesothelioma were for cancer of the larynx (30%), pharynx (23%), and lung (19%) (Tables S5 and S6). Even these estimates do not include some widespread and therefore potentially very important occupational exposures such as UV exposure from sunlight (associated with skin cancers) shift work (associated with breast cancer) and the herbicide glyphosate and insecticides malathion and diazinon (associated with non-Hodgkin's lymphoma). For example, in the UK Burden of Cancer study, breast cancers arising from shift work were estimated to be responsible for over 50% of the female cancer burden arising from occupational exposures[25]. As further epidemiological evidence becomes available, and molecular epidemiologic studies provide insights into the carcinogenic mechanisms of Group 2A exposures (as was the case, for example, with trichloroethylene[26,27] and diesel[28]), IARC's classifications are likely to be re-evaluated. Also, the GBD decisions regarding exclusion are likely to be modified for some of these 2A exposures, potentially increasing the burden attributed to occupational exposures. Finally, no account is made of the apparent causal association between COPD, which can be related to occupational exposures, and lung cancer[29]).

<u>Cancers</u>

Cancer deaths occurred mainly in men and in persons aged 55 to 79 years. Rates in men were about three to six times higher than in women for all but the youngest ages, and rates increased with age. Men are more likely to be employed in tasks that entail exposure to carcinogens and probably more likely to undertake tasks that result in exposure to higher levels of carcinogens[22,30]. In addition, due to latency and the fact that cancer risk increases with age, most people are retired before they develop cancer as a result of work exposures, which explains the high rates at older ages.

Lung cancer was the predominant type of cancer, responsible for about 300,000 deaths. Malignant mesothelioma (primarily pleural in origin) was responsible for another 27,500 deaths. These results reflect the fact that the respiratory tract, and the lung in particular, is the primary route of entry for all airborne carcinogens, and that airborne exposure is the main route of occupational exposure to carcinogens.

. *Occup Environ Med* 2020; 77:151–159. doi: 10.1136/oemed-2019-106012

Changes over time (Tables 3 and S4) and differences between regions (Figures 1 and S2) are affected by changes and differences in exposure to occupational carcinogens. For lung cancer they are, however, also affected by time trends or regional differences in background rates due to varying prevalence of tobacco smoking. For non-asbestos carcinogens, the changes over time reflect changes in employment distribution between different industries and the resultant different probability of exposure. There was insufficient information available to allow differences in exposure probability or level over time to be taken into account in this analysis but it is hoped this will be possible in future analyses.

Comparison with other studies

The CRA 2000 study was the first attempt to comprehensively assess the contribution of occupational carcinogens to cancer[31], but it considered a smaller number of carcinogens and cancer sites than were considered here. The CRA 2000 study estimated that in 2000 there were 152,000 deaths from cancer, compared to the 349,000 estimated for the current study. Looking only at the cancer outcomes included in the CRA 2000 study, the current study estimated about 198,000 (190%) more deaths from lung cancer, 15,000 (36%) fewer deaths from malignant mesothelioma, and 4,500 (64%) fewer deaths from leukaemia. The main reasons for the differences appear to be that in this study additional exposures and outcomes were included (and IARC Group 2A exposures included in the CRA 2000 study were not included), many of the risk measures were different (due to updates in the literature), the estimates were made for the 2016 world population rather than the 2000 population, and the approach to estimating the population at risk was more sophisticated in the current study than was possible for the CRA 2000 study. The differences in the estimates for mesothelioma additionally arise from the different methodologies used in the two studies to develop these estimates.

Other global or national estimates of burden arising from occupational carcinogenic exposures have been published. The most recent and comprehensive of these is that by Rushton and colleagues, who estimated the burden arising from exposure to occupational carcinogens in Britain, providing PAF estimates of 8.2% for men and 2.3% for women for deaths from cancer[25,32]. The higher PAF estimates compared to those in

the current study arise primarily from the inclusion in that study of a much wider range of carcinogens and cancer types, including most relevant IARC Group 2A exposures, and some outcomes with limited epidemiological evidence regarding causality. This is the general approach used in several recent occupational cancer burden and exposure studies[22,33-38].

Methodological considerations and limitations

Methodological issues relevant to the overall study are considered in detail in the occupational risk factors overview paper[39]. The main aspects relevant to the carcinogen analysis are considered in more detail here.

Assumptions regarding latency, turnover, and at-risk period were made when estimating the population at risk. There is reasonable evidence to support these assumptions, but there is a general lack of information on the latency of specific cancers and uncertainty about variation in turnover worldwide, and the risk functions are likely to be complex and not known for most exposure-cancer pairs.

CAREX provided estimates of carcinogen exposure prevalence. That study was focussed on 1990–1993 for Western Europe (and incorporated estimates from North America). It thus specifically reflects exposure circumstances from that time and those regions. This has the advantage that it reflects exposures in the past rather than in the present, which is appropriate for the current analysis due to latency considerations. However, the information is not separately available for different sexes, different ages, or other regions. The logic of the approach used was that in terms of chemical substances, the type of carcinogens to which workers would be exposed are likely to be reasonably consistent within a given industry regardless of the country involved. Similarly, it was assumed the proportion of workers with any exposure is likely to be reasonably consistent within a given industry regardless of the country involved. It appears likely that for most carcinogens, exposures would have been less well controlled over the relevant exposure period in LMI countries than in high-income countries (for example, see references[1-7,40]). To attempt to take this into account, the same exposure prevalences were used for all regions, but in LMI countries a higher proportion of the exposed persons were assumed to be exposed at a high level than was the case in high-income

countries. For most carcinogens, the relative risk used for low exposure was one. This means that the low-exposed persons were considered the same as those who were not occupationally exposed, giving a zero attributable burden, and that in practice the burden for these risk factors was based only on persons with high exposure.

The method used for estimation of relative risk for lung cancer from asbestos exposure required an estimate of the absolute cumulative exposure level for asbestos. This in turn required estimates of years of exposure, and of the levels of exposure during those years. With few published data to provide guidance, the developed method estimated the number of years of work, based on age, using cohort modelling. The estimates of lung cancer deaths were developed using exposure prevalences estimated using the AIR approach, which is based on mesothelioma rates. Since the latency for mesothelioma is longer than for lung cancer, our approach may overestimate current lung cancer risk for countries in which asbestos use declined long before 1990–1993 (e.g., Sweden[41]). The same issue arises for burden from laryngeal and ovarian cancer due to asbestos exposure.

The relative risk estimates came primarily from working cohorts in high-income countries, from a range of time periods, involving a range of exposures and varying follow-up. Therefore, some mismatch between the relative risk estimates used and the exposure circumstances to which they have been applied is likely. Nevertheless, the measures used were considered the most appropriate available.

The prolonged period of risk following exposure for most included carcinogens suggests that a considerable number of previous workers aged 80 and over would remain at risk. However, for non-asbestos exposures, RRs were set to 1.0 for ages 80 and over. This is likely to have led to a moderate underestimate of burden arising from non-asbestos-related cancers (for example, 34% of all asbestos-related cancer deaths were in persons 80 years or older, although the longer latency of asbestos-related cancers suggests the underestimate for other cancers would be lower than this). The curtailing of risk at age 80 arose from a programming error and will be corrected in subsequent GBD iterations.

. *Occup Environ Med* 2020; 77:151–159. doi: 10.1136/oemed-2019-106012

Our study estimates for high-income countries appear to have reasonable agreement with published data on mesothelioma occurrence[42,43]. Nevertheless, there are reasons to consider that the estimates of mesothelioma in the current study are low for some LMI regions, particularly South Asia and China. The all-cause estimates of cause of death are heavily influenced by reported cases. In the relevant countries, there is evidence that mesothelioma is considerably underreported, notwithstanding that there may be an effect from competing causes, with a larger proportion of the population dying at a younger age, before mesothelioma has developed, than occurs in high-income countries. There is no reliable independent information on mesothelioma numbers in these regions to allow comparison to the estimates in this study, and much of the asbestos use probably occurred more recently than in many other countries[44]; however, recent estimates based on asbestos consumption suggest our estimates are lower than would be expected[42]. Balancing this somewhat is the fact that the PAF calculation for mesothelioma assumes that all mesothelioma is a product of occupational asbestos exposure, which could potentially overestimate burden due to occupational asbestos exposure in populations with high non-occupational asbestos exposure.

Finally, no explicit account is made of possible interactions between risk factors in people exposed to multiple risk factors, such as that known to occur in persons exposed to both asbestos and tobacco smoke[45]. The data were not available to allow potential synergistic (or antagonistic) interactions to be included in the estimates, and to the extent these occur, the presented results would probably be underestimates.

. *Occup Environ Med* 2020; 77:151–159. doi: 10.1136/oemed-2019-106012

Table S1 Number of workers exposed per 100,000 workers – risk factor by industry[1]

Risk factor	Agriculture, hunting, forestry, and fishing	Mining and quarrying	Manufacturing	Electricity, gas, and water	Construction	Wholesale and retail trade and restaurants and hotels	Transport, storage, and communication	Financing, insurance, real estate, and business services	Community, social, and personal services
**Arsenic**	54	72	399	148	134	6	-	2	11
**Asbestos**	1,248	10,248	589	1,702	5,203	292	684	16	286
**Benzene**	59	197	308	91	75	1,037	520	41	2,330
**Beryllium**	-	55	207	70	4	2	11	-	3
**Cadmium**	-	-	486	287	291	2	65	-	48
**Chromium VI**	-	346	2,061	409	237	17	369	-	227
**Diesel engine exhaust**	646	21,970	1,192	3,359	5,816	485	13,432	-	920
**Second-hand smoke**	2,082	163	5,249	6,172	4,830	9,278	6,965	4,584	3,633
**Formaldehyde**	186	255	2,103	28	545	53	23	22	594
**Nickel**	-	2,025	1,663	352	47	7	3	-	43
**Polycyclic aromatic hydrocarbons-**	1,021	1,650	3,066	1,328	106	905	-	388	
**Silica**	372	23,049	2,316	1,415	18,860	17	476	2	60
**Strong inorganic acid mist**	-	366	1,488	928	577	264	255	81	189

1: Based on CAREX[15]

. *Occup Environ Med* 2020; 77:151–159. doi: 10.1136/oemed-2019-106012

Table S2 Exposure-outcome pairs for occupational carcinogens, relative risks (95% CI), source and study type

Exposure	Cancer site/type	RR-high[1]	95% CI[2]	RR-low	95% CI	Source of evidence	Study type[3]
Arsenic	Lung	2.05	1.43-2.85	1		Lee-Feldstein 1986[46a]	KS
Asbestos	Larynx	1.38	1.17-1.60	1		IOM, 2006[47b]	M-A
Asbestos (males)	Lung	2.27	1.67-2.85	1.65	1.50-1.79	Lenters et al, 2011[12c]	M-A
Asbestos (females)	Lung	1.86	1.56-21.5	1.52	1.46-1.58	Lenters et al, 2011[12c]	M-A
Asbestos	Mesothelioma[4]						
Asbestos	Ovary	1.77	1.37-2.28	1		Camargo et al, 2011[48]	M-A
Benzene	Leukaemia	2.62	1.57-4.39	1.64	1.13-2.39	Khalade et al, 2010[49]	M-A
Beryllium	Lung	1.17	1.08-1.28	1		Schubauer-Berigan et al, 2011[50e]	KS
Cadmium	Lung	1.19	1.09-1.29	1		Verougstraete et al, 2003[51f]	M-A
Chromium VI	Lung	1.18	1.12-1.25	1		Cole & Rodu, 2005[52]	M-A
Diesel engine exhaust	Lung	1.47	1.29-1.67	1		Lipsett & Campelman, 1999[16]	M-A
Second-hand smoke	Breast	1.07	1.02-1.13	1		Chen et al, 2014[53]	M-A
Second-hand smoke	Lung	1.24	1.18-1.29	1		Stayner et al, 2007[54]	M-A
Formaldehyde	Leukaemia	1.47	1.19-1.83	1		Collins and Lineker, 2004[55]	M-A
Formaldehyde	Nasopharynx[5]	2.1	1.05-4.21	1		Hauptmann et al, 2004[56g]	KS
Nickel	Lung	2.1	1.3-3.2	1		Grimsrud et al 2005[57h]	KS
PAHs	Lung	1.31	1.16-1.48	1		Armstrong et al, 2004[58]	M-A
Silica	Lung	1.70	1.23-2.34	1.54	1.16-2.05	Liu et al, 2013[59i]	KS
Strong inorganic-acid mists	Larynx	4.28	2.13-8.58	1.91	0.97-3.78	Soskolne et al, 1992[60j]	KS
Trichloroethylene	Kidney	1.24	1.06-1.45	1		Kelsh et al, 2010[61]	M-A

1: RR=Relative risk

2: 95% confidence interval.

3: M-A = meta-analysis; KS = key study.

4: The population attributable risk PAF for mesothelioma was calculated directly from the excess mesothelioma rate divided by the overall mesothelioma rate.

5: Nasal cavity and paranasal sinuses.

. *Occup Environ Med* 2020; 77:151–159. doi: 10.1136/oemed-2019-106012

Notes:

a: Although later updates have been carried out by Lubin et al, 2000[62], there is uncertainty about the dose-response curve. Therefore the Lee-Feldstein 1986[46] paper remains the single key study in the area.

b: Meta-analysis based on review by IOM, 2006[47], using inverse weighted average from relevant occupational cohorts included in that report.

c: Final relative risks used based on the meta-analysis using the five best studies and estimated exposure of 2.0 times the US PEL (high) and 0.5 times the US PEL (low) for males and 1.0 times the US PEL (high) and 0.2 times the US PEL (low) for females.

d: RRs based on reported data in this study.

e: This single study has all the main occupational cohorts exposed to beryllium in an occupational setting. It is therefore essentially a pooled analysis of all relevant studies.

f: Meta-analysis based on review by Verougstraete et al, 2003[51], using inverse weighted average from seven occupational cohorts included in that study.

g: Cited as the key defining evidence by IARC[63].

h: The RR from Grimsrud et al, 2005[57] is for any exposure to water soluble nickel and is adjusted for smoking, other exposures such as arsenic, asbestos, strong inorganic acid mist, cobalt, and also exposures outside the refinery. It is therefore considered the key study in this area.

i: This is the key study as it provides detailed information on cumulative silica exposure in a large cohort with good control of smoking.

j: This is the key study because the other only other potentially relevant studies (Steenland[64] and Soskolne et al, 1984[65]) were of acid mists experienced by chemical workers involved in metal processing and the exposures were thus relatively high. The lower limit of the RR for low exposure was 0.97, which was rounded to 1.0 for the analysis.

. *Occup Environ Med* 2020; 77:151–159. doi: 10.1136/oemed-2019-106012

Table S3 Occupational-attributable deaths due to carcinogens by risk factor and region, 2016, percent

Risk factor[1]	Number	Arsenic	Asbes	Benzene	Beryl	Cadm	Chrom	DEE	Form	Nickel	PAH	SHS	Silica	SIAM	Trich	Total
High-income North America	**56,167**	1.2	79.2	0.2	0.0	0.0	0.1	1.5	0.0	1.0	0.3	12.1	7.5	0.2	0.0	100.0
Australasia	**5,167**	0.9	88.4	0.2	0.0	0.0	0.0	0.7	0.0	0.7	0.2	8.5	4.5	0.1	0.0	100.0
High-income Asia Pacific	**23,301**	1.5	78.1	0.2	0.0	0.0	0.1	1.1	0.0	1.3	0.3	12.1	8.7	0.1	0.0	100.0
Western Europe	**92,443**	1.0	88.1	0.2	0.0	0.0	0.1	0.7	0.0	0.8	0.2	6.9	5.5	0.2	0.0	100.0
Southern Latin America	**3,492**	2.2	58.8	0.8	0.1	0.2	0.5	8.0	0.3	2.3	1.9	15.1	13.9	1.1	0.1	100.0
Eastern Europe	**10,462**	2.2	62.6	0.5	0.0	0.1	0.1	1.9	0.1	2.1	0.4	15.7	15.5	1.3	0.0	100.0
Central Europe	**10,478**	3.0	59.7	0.3	0.0	0.1	0.2	1.9	0.0	2.6	0.5	17.3	17.4	1.0	0.0	100.0
Central Asia	**1,597**	2.7	34.6	1.7	0.1	0.3	0.6	11.8	0.6	3.4	2.4	21.1	22.9	2.5	0.1	100.0
Central Latin America	**3,643**	2.7	48.0	2.8	0.1	0.3	0.6	9.8	1.1	2.7	2.2	16.1	15.8	2.1	0.1	100.0
Andean Latin America	**872**	2.7	46.8	2.7	0.1	0.3	0.6	10.7	1.0	2.8	2.2	16.2	16.5	1.4	0.1	100.0
Caribbean	**1,301**	3.1	34.4	1.3	0.1	0.3	0.7	10.5	0.5	3.1	2.7	27.0	17.3	3.3	0.0	100.0
Tropical Latin America	**6,774**	2.1	50.7	1.1	0.1	0.2	0.5	7.5	0.4	2.2	1.8	22.7	12.9	2.5	0.0	100.0
East Asia	**80,266**	4.7	27.1	0.6	0.2	0.5	0.9	11.4	0.6	5.0	3.3	24.1	25.9	1.0	0.0	100.0
Southeast Asia	**15,762**	3.2	43.2	1.1	0.1	0.3	0.7	10.6	0.8	3.5	2.5	15.6	21.0	1.5	0.0	100.0
Oceania	**127**	2.4	46.6	2.4	0.1	0.3	0.5	9.7	1.0	2.8	2.0	15.3	18.3	1.6	0.0	100.0
North Africa and Middle East	**11,822**	2.0	62.8	1.4	0.1	0.2	0.4	7.1	0.6	2.3	1.6	10.2	14.8	1.4	0.0	100.0
South Asia	**19,116**	2.6	47.4	1.5	0.1	0.3	0.5	8.8	0.9	3.0	2.0	10.9	19.4	6.5	0.0	100.0
Southern sub-Saharan Africa	**2,257**	0.7	85.7	0.6	0.0	0.1	0.2	2.6	0.2	0.8	0.6	5.0	5.2	0.6	0.0	100.0
Western sub-Saharan Africa	**1,369**	2.4	35.1	3.5	0.1	0.2	0.5	9.0	1.3	2.8	1.9	23.7	18.0	3.4	0.1	100.0
Eastern sub-Saharan Africa	**1,826**	2.2	49.2	2.6	0.1	0.2	0.4	7.4	1.7	2.5	1.6	14.3	15.8	4.6	0.1	100.0
Central sub-Saharan Africa	**498**	1.7	58.5	1.5	0.1	0.2	0.4	7.9	0.6	2.3	1.4	10.3	14.6	2.6	0.0	100.0
High SDI[2]	**178,393**	1.2	83.2	0.2	0.0	0.0	0.1	1.1	0.0	1.0	0.3	9.5	6.8	0.2	0.0	100.0
High-middle SDI	**59,640**	3.4	48.2	0.7	0.1	0.3	0.6	6.9	0.5	3.3	2.0	18.9	18.7	1.1	0.0	100.0
Middle SDI	**76,190**	3.8	34.5	0.9	0.2	0.4	0.8	10.7	0.6	4.2	2.7	21.3	23.1	1.5	0.0	100.0
Low-middle SDI	**30,235**	3.0	44.0	1.2	0.1	0.3	0.6	9.5	0.8	3.4	2.2	14.0	20.6	4.2	0.0	100.0
Low SDI	**4,283**	2.3	47.4	2.2	0.1	0.2	0.5	8.4	1.2	2.9	1.8	12.8	19.1	4.1	0.0	100.0
Global	**348,741**	2.3	62.7	0.5	0.1	0.2	0.4	5.0	0.3	2.3	1.3	14.1	13.8	1.0	0.0	100.0

1: Asbes=asbestos; Beryl=beryllium; Cadm=cadmium; Chrom=chromium; DEE=diesel engine exhaust; Form=formaldehyde; PAH=polycyclic aromatic hydrocarbons; SHS=second-hand smoke; SIAM=strong inorganic acid mist; Trich=trichloroethylene

2: SDI=Socio-demographic index

Table S4 Change in occupation-attributable deaths due to carcinogens, 1990 and 2016, number and rate (per 100,000 persons), by region

	Deaths per 100,000 persons			DALYs per 100,000 persons		
**Region**	**1990**	**2016**	**% change**	**1990**	**2016**	**% change**
High-income North America	41.2	33.4	-18.9	802	573	-28.5
Australasia	42.6	38.6	-9.4	828	656	-20.8
High-income Asia Pacific	14.0	22.5	61.3	269	358	33.0
Western Europe	43.7	41.1	-5.9	865	720	-16.9
Southern Latin America	13.4	13.5	1.2	323	292	-9.6
Eastern Europe	14.4	10.4	-27.7	372	247	-33.6
Central Europe	14.9	18.3	23.0	391	424	8.4
Central Asia	8.7	5.6	-35.3	246	150	-38.9
Central Latin America	4.7	4.6	-3.7	114	107	-6.3
Andean Latin America	7.5	4.8	-36.2	161	102	-36.7
Caribbean	7.0	7.8	11.6	164	182	11.5
Tropical Latin America	10.2	8.9	-12.9	201	194	-3.3
East Asia	9.5	13.2	39.8	250	321	28.7
Southeast Asia	6.0	7.6	26.1	144	178	23.5
Oceania	4.2	4.7	12.7	111	121	9.4
North Africa and Middle East	9.1	7.7	-15.9	216	175	-18.9
South Asia	2.7	3.9	41.0	71	93	31.6
Southern sub-Saharan Africa	13.1	11.5	-12.7	304	242	-20.3
Western sub-Saharan Africa	2.1	1.8	-14.0	54	47	-13.4
Eastern sub-Saharan Africa	2.4	2.5	6.4	60	61	2.5
Central sub-Saharan Africa	2.5	2.4	-5.7	61	56	-8.7
High SDI[2]	36.4	34.0	-6.5	718	589	-17.9
High-middle SDI	13.7	12.5	-8.6	340	289	-15.1
Middle SDI	6.9	9.0	29.8	177	216	22.0
Low-middle SDI	4.0	5.2	29.9	99	123	24.3
Low SDI	2.6	2.9	8.9	66	71	7.1
Global	15.0	13.5	-9.8	320	272	-14.8

1: SDI=Socio-demographic index

. *Occup Environ Med* 2020; 77:151–159. doi: 10.1136/oemed-2019-106012

Table S5 Occupation-attributable cancer deaths, 2016, by carcinogen and cancer type, including Group 2A exposures and outcomes with "limited" evidence, number and percent

Carcinogen	Deaths	%
Arsenic[1]	8,073	1.8
Asbestos	227,764	51.1
Colon	1,105	
Larynx	3,7435	
Lung	181,450	
Mesothelioma	27,612	
Ovary	6,022	
Pharynx	341	
Stomach	7,490	
Benzene[2]	1,899	0.4
Beryllium[1]	259	0.1
Cadmium	759	0.2
Lung	605	
Kidney	154	
Chromium[1]	1,276	0.3
Cobalt with tungsten[1]	8,153	0.8
Diesel engine exhaust	19,670	1.8
Bladder	2,170	
Lung	17,500	
Ethylene oxide[2]	14	0.0
Formaldehyde	1,056	0.2
Leukaemia	608	
Nasopharynx cancer	448	
Lead	10,406	2.3
Lung	4,631	
Stomach	5,775	
Nickel[1]	8,101	1.8
PAH	5,549	1.2
Bladder	1,023	
Lung	4,526	
Second-hand smoke	104,436	23.4
Breast	4,864	
Larynx	28,212	
Lung	44,382	
Pharynx	26,979	
Silica[1]	47,999	10.8
Strong inorganic-acid mists	9,524	2.1
Larynx	3,535	
Lung	5,989	
Tetrachloroethylene	8,981	2.0
Cervix	2,016	
Oesophagus	6,964	

Trichloroethylene	1,164	0.3
Liver	1,040	
Kidney	58	
NHL	66	
Total	445,560	100.0

1: Causes lung cancer
2: Causes leukaemia

. *Occup Environ Med* 2020; 77:151–159. doi: 10.1136/oemed-2019-106012

Table S6 Occupation-attributable cancer deaths and PAFs, 2016, by cancer type and carcinogen, including Group 2A exposures and outcomes with "limited" evidence, number and percent

Cancer type	Deaths	%	PAF
Bladder cancer	3,181	0.7	1.7
Diesel engine exhaust	*2,170*		
Polycyclic aromatic hydrocarbons	*1,023*		
Cervix cancer[1]	2,016	0.5	0.8
Colon cancer[2]	1,105	0.2	0.1
Kidney cancer	212	0.0	0.2
Cadmium	*154*		
Trichloroethylene	*58*		
Larynx cancer	33,596	7.5	30.3
Asbestos	*3,743*		
Second-hand smoke	*28,212*		
Strong inorganic-acid mists	*3,535*		
Leukaemia	2,481	0.6	0.8
Benzene	*1,899*		
Formaldehyde	*608*		
Liver cancer[3]	1,040	0.2	0.1
Lung cancer	315,487	70.8	18.5
Arsenic	*8,073*		
Asbestos	*181,450*		
Beryllium	*259*		
Cadmium	*605*		
Chromium	*1,276*		
Cobalt (with tungsten)	*8,153*		
Diesel engine exhaust	*17,500*		
Environmental tobacco smoke	*44,382*		
Lead	*4,631*		
Nickel	*8,101*		
Polycyclic aromatic hydrocarbons	*4,526*		
Silica	*47,999*		
Strong inorganic-acid mists	*5,989*		
Mesothelioma[2]	27,607	6.2	91.4
Nasopharynx cancer[4]	448	0.1	0.7
Oesophagus cancer[1]	6964	1.6	1.7
Ovary cancer[2]	6,022	1.4	3.6
Pharynx cancer	27,242	6.1	23.0
Asbestos	*341*		
Second-hand smoke	*26,979*		
NHL	80	0.0	0
Ethylene oxide	*14*		
Trichloroethylene	*66*		
Stomach cancer	13,214	3.0	1.6
Asbestos	*7,490*		
Lead	*5,775*		
Total	445,560	100.0	5.0

1: Caused by tetrachloroethylene
2: Caused by asbestos
3: Caused by trichloroethylene
4: Caused by formaldehyde

. *Occup Environ Med* 2020; 77:151–159. doi: 10.1136/oemed-2019-106012

Figure S1 Occupation-attributable cancer deaths and DALYs by age and sex, 2016 (per 100,000 persons)

Figure S1a Occupation-attributable cancer deaths, 2016 (per 100,000 persons)

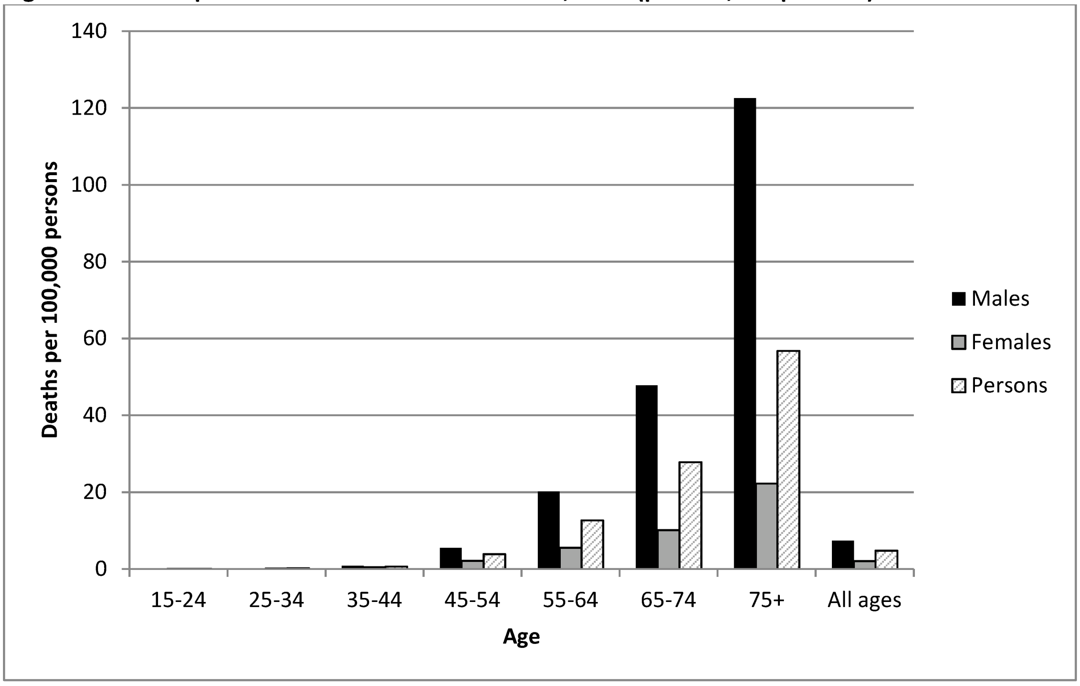

Figure S1b Occupation-attributable cancer DALYs, 2016 (per 100,000 persons)

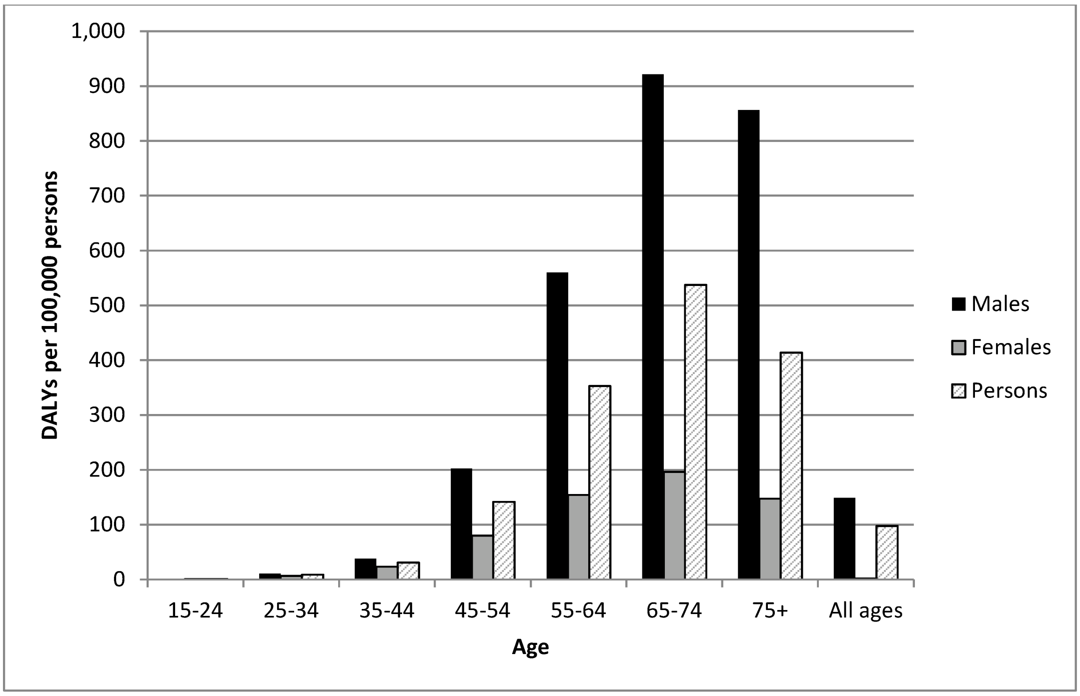

DALY=Disability-adjusted life year.

Figure S2 Occupation-attributable cancer DALYs, by region, 2016 (per 100,000 persons)*

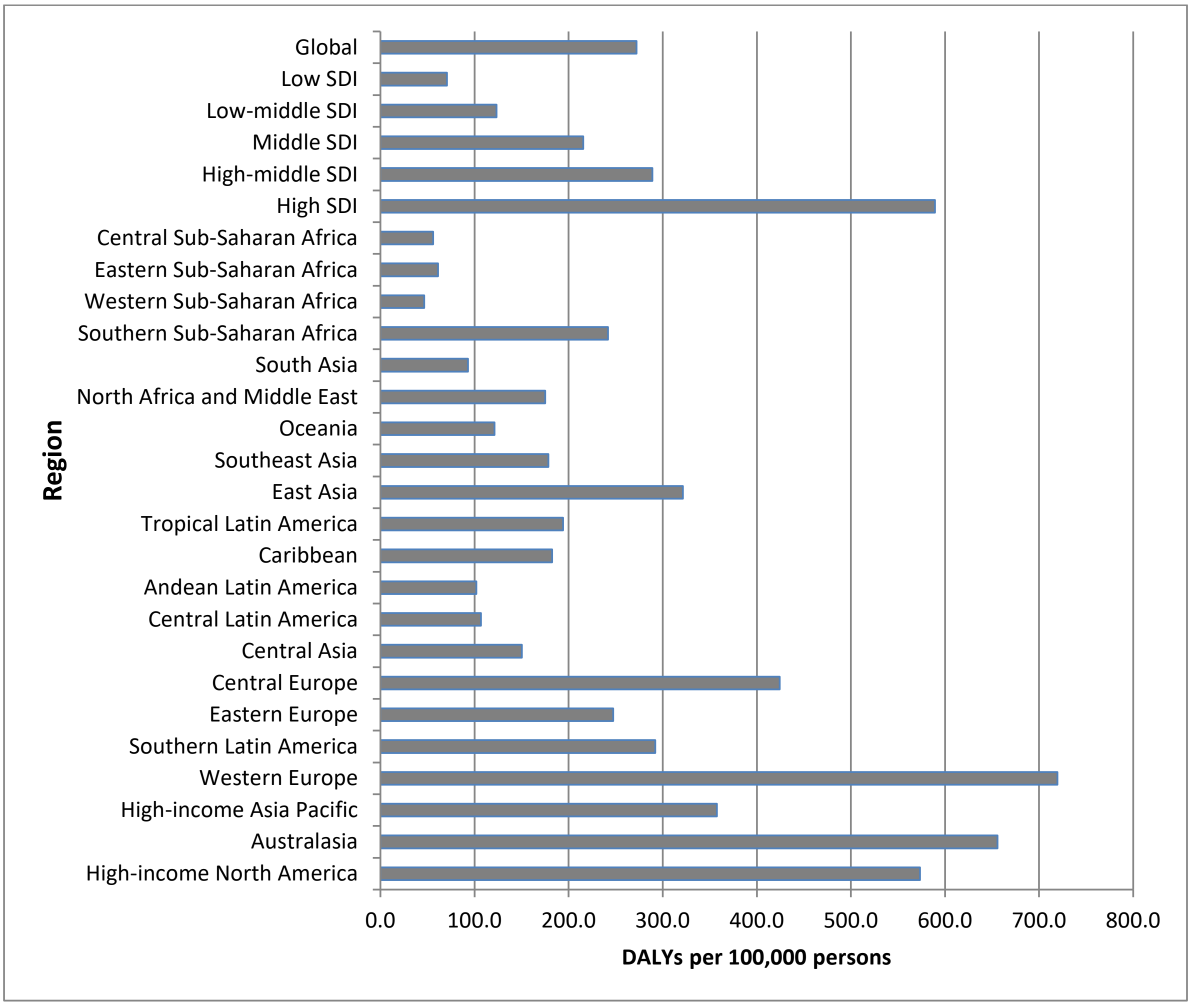

* Age-standardised
DALY=Disability-adjusted life year.

. *Occup Environ Med* 2020; 77:151–159. doi: 10.1136/oemed-2019-106012

REFERENCES FOR SUPPLEMENTARY MATERIAL

1. Dosemeci M, McLaughlin J, Chen J, et al. Historical total and respirable silica dust exposure levels in mines and pottery factories in China. *Scandinavian Journal of Work, Environment and Health.* 1995;21(Suppl 2):39–43.

2. Partanen T, Jaakkola J, Tossavainen A. Silica, silicosis and cancer in Finland. *Scandinavian Journal of Work, Environment and Health.* 1995;21(Suppl 2):84–86.

3. Myers J, Lewis P, Hofmeyr W. Respiratory health of brickworkers in Cape Town, South Africa: Background, aims, and dust exposure determinations. *Scandinavian Journal of Work, Environment and Health.* 1989;15(3):180–187.

4. National Institute for Occupational Safety and Health (NIOSH). *Work-related Lung Disease Surveillance Report 1999.* Cincinnati: U.S. Department of Health and Human Services, National Institute for Occupational Safety and Health;1999. DHHS (NIOSH) Number 2000-105.

5. National Institute for Occupational Safety and Health (NIOSH). *Injuries, illnesses, and hazardous exposures in the mining industry, 1986-1995: A surveillance report.* Washington, DC: U.S. Department of Health and Human Services, National Institute for Occupational Safety and Health.;2000.

6. Rees D, Cronje R, du Toit R. Dust exposure and pneumoconiosis in a South African pottery. 1. Study objectives and dust exposure. *British Journal of Industrial Medicine.* 1992;49(7):459–464.

7. Yin S, Li Q, Liu Y, Tian F, Du C, Jin C. Occupational exposure to benzene in China. *British Journal of Industrial Medicine.* 1987;44(3):192–195.

8. Nelson D, Concha-Barrientos M, Driscoll T, et al. The global burden of selected occupational diseases and injury risks: methodology and summary. *American Journal of Industrial Medicine.* 2005;48:400-418.

9. Hutchings S, Rushton L. Occupational cancer in Britain: statistical methodology. *British Journal of Cancer.* 2012;107:S8-S17.

10. Cherrie J, Van Tongeren M, S S. Exposure to occupational carcinogens in Great Britain. *Annals of Occupational Hygiene.* 2007;51(8):653-664.

11. Steenland K, Armstrong B. An overview of methods for calculating the burden of disease due to specific risk factors. *Epidemiology.* 2006;17(5):512-519.

12. Lenters V, Vermeulen R, Dogger S, et al. A meta-analysis of asbestos and lung cancer: is better quality exposure assessment associated with steeper slopes of the exposure-response relationships? *Environmental Health Perspectives.* 2011;119(11):1547-1555.

13. International Agency for Research on Cancer. *IARC Monographs on the Evaluation of Carcinogenic Risks to Humans. Volume 105. Diesel and Gasoline Engine Exhausts and Some Nitroarenes.* Geneva: World Health Organization;2013.

14. Laborsta Internet. ILO; 2014. laborsta.ilo.org. Accessed 2009-2012.

15. Kauppinen T, Toikkanen J, Pedersen D, et al. Occupational exposure to carcinogens in the European Union. *Occupational and Environmental Medicine.* 2000;57(1):10-18.

. *Occup Environ Med* 2020; 77:151–159. doi: 10.1136/oemed-2019-106012

16. Lipsett M, Campleman S. Occupational exposure to diesel exhaust and lung cancer: a meta-analysis. *American Journal of Public Health.* 1999;89(7):1009-1017.

17. Jaakkola M, Jaakkola J. Impact of smoke-free workplace legislation on exposures and health: possibilities for prevention. *European Respiratory Journal.* 2006;28(2):397-408.

18. McNabola A, Gill L. The control of environmental tobacco smoke: a policy review. *International Journal of Environmental Research and Public Health.* 2009;6(2):741-758.

19. World Health Organization (WHO). *WHO report on the global tobacco epidemic, 2017: Monitoring tobacco use and prevention policies.* Geneva2017.

20. Gu D, Wu X, Reynolds K, et al. Cigarette smoking and exposure to environmental tobacco smoke in China: the international collaborative study of cardiovascular disease in Asia. *American Journal of Public Health.* 2004;94(11):1972-1976.

21. CAREX Canada. Surveillance of environmental and occupational exposures for cancer prevention. 2012; http://www.carexcanada.ca/en/. Accessed May, 2014.

22. Carey R, Driscoll T, Peters S, et al. Estimated prevalence of exposure to occupational carcinogens in Australia (2011-2012). *Occupational and Environmental Medicine.* 2014;71(1):55-62.

23. International Agency for Research on Cancer. *IARC Monographs on the Evaluation of Carcinogenic Risks to Humans. Volume 68. Silica, some silicates, Coal Dust and para-aramid fibrils.* Geneva: World Health Organization;1997.

24. Vermeulen R, Silverman D, Garshick E, Vlaanderen J, Portengen L, Steenland K. Exposure-response estimates for diesel engine exhaust and lung cancer mortality based on data from three occupational cohorts. *Environmental Health Perspectives.* 2014;122(2):172-177.

25. Rushton L, Hutchings S, Fortunato L, et al. Occupational cancer burden in Great Britain. *British Journal of Cancer.* 2012;107(Suppl 1):S3-S7.

26. Hosgood H, Zhang L, Tang X, et al. Decreased numbers of CD4(+) Naive and Effector Memory T Cells, and CD8(+) Naïve T Cells, are associated with trichloroethylene exposure. *Frontiers in Oncology.* 2012;1:53.

27. Vermeulen R, Zhang L, Spierenburg A, et al. Elevated urinary levels of kidney injury molecule-1 among Chinese factory workers exposed to trichloroethylene. *Carcinogenesis.* 2012;33(8):1538-1541.

28. Lan Q, Vermeulen R, Dai Y, et al. Occupational exposure to diesel engine exhaust and alterations in lymphocyte subsets. *Occupational and Environmental Medicine.* 2015;72(5):354-359.

29. Durham A, Adcock I. The relationship between COPD and lung cancer. *Lung Cancer.* 2015;90(2):121-127.

30. ILOSTAT database. ILO; 2015. http://www.ilo.org/ilostat. Accessed 2012-2017.

31. Driscoll T, Nelson D, Steenland K, et al. The global burden of disease due to occupational carcinogens. *American Journal of Industrial Medicine.* 2005;48(6):419-431.

32. Rushton L, Bagga S, Bevan R, et al. Occupation and cancer in Britain. *British Journal of Cancer.* 2010;102(9):1428-1437.

33. Azevedo ESG, de Moura L, Curado MP, et al. The Fraction of Cancer Attributable to Ways of Life, Infections, Occupation, and Environmental Agents in Brazil in 2020. *PLoS One.* 2016;11(2):e0148761.

34. Fritschi L, Driscoll T. Cancer due to occupation in Australia. *Australian and New Zealand Journal of Public Health.* 2006;30(3):213-219.

35. Marant Micallef C, Shield KD, Vignat J, et al. Cancers in France in 2015 attributable to occupational exposures. *International journal of hygiene and environmental health.* 2019;222(1):22-29.

36. Nurminen M, Karjalainen A. Epidemiologic estimate of the proportion of fatalities related to occupational factors in Finland. *Scandinavian Journal of Work, Environment and Health.* 2001;27(3):161-213.

37. Takala J, Hämäläinen P, Saarela K, et al. Global estimates of the burden of injury and illness at work in 2012. *Journal of Occupational and Environmental Hygiene.* 2014;11(5):326-337.

38. Takala J, Urrutia M, Hämäläinen P, Saarela K. The global and European work environment – numbers, trends, and strategies. *Scandinavian Journal of Work, Environment and Health.* 2009;Supplement 7:15-23.

39. GBD 2016 Occupational Risk Factors Collaborators. Global and regional burden of disease and injury arising from occupational exposures in 2016: a systematic analysis for the Global Burden of Disease Study 2016. *Occupational and Environmental Medicine.* 2019:Submitted.

40. Shendell D, Noomnual S, Chishti S, Sorensen Allacci M, Madrigano J. Exposures resulting in safety and health concerns for child laborers in less developed countries. *Journal of Environmental and Public Health.* 2016:2016:3985498.

41. Järvholm B, Burdorf A. Emerging evidence that the ban on asbestos use is reducing the occurrence of pleural mesothelioma in Sweden. *Scandinavian Journal of Public Health.* 2015;43(8):875-881.

42. Odgerel C, Takahashi K, Sorahan T, et al. Estimation of the global burden of mesothelioma deaths from incomplete national mortality data. *Occupational and Environmental Medicine.* 2017;74(12):851-858.

43. Park E, Takahashi K, Hoshuyama T, et al. Global magnitude of reported and unreported mesothelioma. *Environmental Health Perspectives.* 2011;119(4):514-518.

44. Dave SK, Beckett WS. Occupational asbestos exposure and predictable asbestos-related diseases in India. *Am J Ind Med.* 2005;48(2):137-143.

45. Ngamwong Y, Tangamornsuksan W, Lohitnavy O, et al. Additive synergism between asbestos and smoking in lung cancer risk: A systematic review and meta-analysis. *PlosOne.* 2015;10(8):e0135798.

46. Lee-Feldstein A. Cumulative exposure to arsenic and its relationship to respiratory cancer among copper smelter employees. *Journal of Occupational Medicine.* 1986;28(4):296-302.

47. Institute of Medicine of the National Academies (IOM). *Asbestos: selected cancers.* Washington, DC: The National Academies Press;2006.

48. Camargo M, Stayner L, Straif K, et al. Occupational exposure to asbestos and ovarian cancer: a meta-analysis. *Environmental Health Perspectives.* 2011;119(9):1211-1217.

49. Khalade A, Jaakkola M, Pukkala E, Jaakkola J. Exposure to benzene at work and the risk of leukemia: a systematic review and meta-analysis. *Environmental Health.* 2010;9:31.

50. Schubauer-Berigan M, Deddens J, Couch J, Petersen M. Risk of lung cancer associated with quantitative beryllium exposure metrics within an occupational cohort. *Occupational and Environmental Medicine.* 2011;68(5):354-360.

51. Verougstraete V, Lison D, Hotz P. Cadmium, lung and prostate cancer: a systematic review of recent epidemiological data. *Journal of Toxicology and Environmental Health B Critical Reviews.* 2003;6(3):227-255.

52. Cole P, Rodu B. Epidemiologic studies of chrome and cancer mortality: a series of meta-analyses. *Regulatory Toxicology and Pharmacology.* 2005;43(3):225-231.

53. Chen C, Huang Y-B, Liu X-O, et al. Active and passive smoking with breast cancer risk for Chinese females: a systematic review and meta-analysis. *Chinese Journal of Cancer.* 2014;33(6):306–316.

54. Stayner L, Bena J, Sasco A, et al. Lung cancer risk and workplace exposure to environmental tobacco smoke. *American Journal of Public Health.* 2007;97(3):545-551.

55. Collins J, Lineker G. A review and meta-analysis of formaldehyde exposure and leukemia. *Regulatory Toxicology and Pharmacology.* 2004;40(2):81-91.

56. Hauptmann M, Lubin J, Stewart P, Hayes R, Blair A. Mortality from solid cancers among workers in formaldehyde industries. *American Journal of Epidemiology.* 2004;159(12):1117-1130.

57. Grimsrud T, Berge S, Haldorsen T, Andersen A. Can lung cancer risk among nickel refinery workers be explained by occupational exposures other than nickel? *Epidemiology.* 2005;16(2):146-154.

58. Armstrong B, Hutchinson E, Unwin J, Fletcher T. Lung cancer risk after exposure to polycyclic aromatic hydrocarbons: a review and meta-analysis. *Environmental Health Perspectives.* 2004;112(9):970-978.

59. Liu Y, Steenland K, Rong Y, et al. Exposure response analysis and risk assessment for lung cancer in relationship to silica exposure: a 44-year cohort study of 34,018 workers. *American Journal of Epidemiology.* 2013;178(9):1424-1433.

60. Soskolne C, Jhangri G, Siemiatycki J, et al. Occupational exposure to sulfuric acid in southern Ontario, Canada, in association with laryngeal cancer. *Scandinavian Journal of Work, Environment and Health.* 1992;18(4):225-232.

61. Kelsh M, Alexander D, Mink P, Mandel J. Occupational trichloroethylene exposure and kidney cancer: a meta-analysis. *Epidemiology.* 2010;21(1):95-102.

62. Lubin J, Pottern L, Stone B, Fraumeni JJ. Respiratory cancer in a cohort of copper smelter workers: results from more than 50 years of follow-up. *American Journal of Epidemiology.* 2000;151(6):554-565.

63. International Agency for Research on Cancer. *IARC Monographs on the Evaluation of Carcinogenic Risks to Humans. Volume 100F. A review of human carcinogens: chemical agents and related occupations.* Geneva: World Health Organization;2012.

64. Steenland K. Laryngeal cancer incidence among workers exposed to acid mists (United States). *Cancer Causes and Contol.* 1997;8(1):34-38.

. *Occup Environ Med* 2020; 77:151–159. doi: 10.1136/oemed-2019-106012

65. Soskolne C, Zeighami E, Hanis N, et al. Laryngeal cancer and occupational exposure to sulfuric acid. *American Journal of Epidemiology.* 1984;120(3):358-369.
