# Supplementary material for: Global and regional burden of cancer in 2016 arising from occupational exposure to selected carcinogens: a systematic analysis for the Global Burden of Disease Study 2016
Source: Occup Environ Med. 2020 Feb 13;77(3):151–9. doi: 10.1136/oemed-2019-106012 (PMC7035689; doi:10.1136/oemed-2019-106012)
Supplement: Supplementary data [file oemed-2019-106012supp002.pdf]

GBD 2016 Occupational Carcinogens Collaborators

Tim Driscoll[1], Lesley Rushton[2], Kyle Steenland[3], Sally Hutchings[4], Kurt Straif[5], Degu Abate[6], Omar Abdel-Rahman[7,8], Dilaram Acharya[9,10], Mohsen Afarideh[11], Fares Alahdab[12], Ziyad Al-Aly[13,14], Mina Anjomshoa[15], Olatunde Aremu[16], Al Artaman[17], Zerihun Ataro[6], Beatriz Paulina Ayala Quintanilla[18,19], Alaa Badawi[20,21], Masoud Behzadifar[22], Meysam Behzadifar[23], Mircea Beuran[24,25], Krittika Bhattacharyya[26,27], Ali Bijani[28], Tone Bjørge[29,30], Zahid A Butt[31,32], Juan J Carrero[33], Félix Carvalho[34,35], Carlos A Castañeda-Orjuela[36,37], Ester Cerin[38,39], Pankaj Chaturvedi[40], Odgerel Chimed-Ochir[41], Raquel Crider[42], Christopher Stephen Crowe[43], Lalit Dandona[44,45], Rakhi Dandona[44,45], Anh Kim Dang[46], Ahmad Daryani[47], Beruk Berhanu Desalegn[48], Samath Dhamminda Dharmaratne[45,49], Shirin Djalalinia[50], Andem Effiong[51], Ziad El-Khatib[52], Alireza Esteghamati[11], Mohammad Fareed[53], Eduarda Fernandes[54], Irina Filip[55,56], Florian Fischer[57], Takeshi Fukumoto[58,59], Silvano Gallus[60], Teklu Gebrehiwo Gebremichael[61], Kebede Embaye Gezae[62], Ayman Grada[63], Andre Luiz Sena Guimaraes[64], Rahul Gupta[65,66], Nima Hafezi-Nejad[67,68], Arvin Haj-Mirzaian[69,70], Arya Haj-Mirzaian[67,69], Randah R Hamadeh[71], Samer Hamidi[72], Hadi Hassankhani[73,74], Simon I. Hay[45,75], Mohamed I Hegazy[76], Andualem Henok[77], Chi Linh Hoang[78], Michael K. Hole[79], H Dean Hosgood[80], Mostafa Hosseini[81], Mihaela Hostiuc[25,82], Sorin Hostiuc[83,84], Seyed Sina Naghibi Irvani[85,86], Sheikh Mohammed Shariful Islam[87,88], Mihajlo Jakovljevic[89], Ravi Prakash Jha[90], Mikk Jürisson[91], Amaha Kahsay[92], Narges Karimi[93,94], Amir Kasaeian[95,96], Zemenu Yohannes Kassa[97], Yousef Saleh Khader[98], Morteza Abdullatif Khafaie[99], Ejaz Ahmad Khan[100], Mohammad Hossein Khosravi[101], Jagdish Khubchandani[102], Aliasghar A Kiadaliri[103], Yun Jin Kim[104], Manolis Kogevinas[235], David Koh[105,106], Soewarta Kosen[107], Ai Koyanagi[108,109], G Anil Kumar[44], Deepesh P Lad[110], Dharmesh Kumar Lal[44], Faris Hasan Lami[111], Arman Latifi[112], James Leigh[113], Shai Linn[114], Marek Majdan[115], Reza Malekzadeh[116,117], Deborah Carvalho Malta[118], Mohammad Ali Mansournia[81], Benjamin Ballard Massenburg[43], Addisu Melese[119], Mulugeta Melku[120], Ziad A Memish[121,122], Walter Mendoza[123], Getnet Mengistu[124,125], Tuomo J Meretoja[126], Tomislav Mestrovic[127,128], Bartosz Miazgowski[129], Tomasz Miazgowski[130], Erkin M Mirrakhimov[131,132], Babak Moazen[133,134], Shafiu Mohammed[135,136], Farnam Mohebi[86,137], Ali H Mokdad[45,75], Yoshan Moodley[138], Mahmood Moosazadeh[139], Ghobad Moradi[140,141], Lidia Morawska[142], Shane Douglas Morrison[143], Seyyed Meysam Mousavi[144], Ghulam Mustafa[145,146], Ionut Negoi[24,147], Ruxandra Irina Negoi[148,149], Cuong Tat Nguyen[46], Trang Huyen Nguyen[78], Dina Nur Anggraini Ningrum[150,151], Molly R Nixon[45], Richard Ofori-Asenso[152,153], Felix Akpojene Ogbo[154], Andrew T Olagunju[155,156], Bolajoko Olubukunola Olusanya[157], Doris D V Ortega-Altamirano[158], Mahesh P A[159], Eun-Kee Park[160], David M. Pereira[54,161], Swayam Prakash[162], Mostafa Qorbani[163], Amir Radfar[164,165], Anwar Rafay[166,167], Alireza Rafiei[168,169], Fakher Rahim[170,171], Vafa Rahimi-Movaghar[172], Fatemeh Rajati[173], Robert C Reiner[45,75], Andre M. N. Renzaho[174], Satar Rezaei[175], Leonardo Roever[176], Mohammad Saadat[177], Basema Saddik[178], Saeed Safari[179], Saeid Safiri[180], Amirhossein Sahebkar[181,182], Mohammad Ali Sahraian[183], Hamideh Salimzadeh[116], Abdallah M. Samy[184], Juan Sanabria[185,186], Rodrigo Sarmiento-Suárez[187], Brijesh Sathian[188,189], David C. Schwebel[190], Sadaf G. Sepanlou[116], Berrin Serdar[191,192], Masood Ali Shaikh[193], Rajesh Sharma[194], Jun She[195], Mika Shigematsu[196], Reza Shirkoohi[197,198], Si Si[199], Dhirendra Narain Sinha[200], Moslem Soofi[201], Jeffrey D Stanaway[45,75], Mark A Stokes[202], Rafael Tabarés-Seisdedos[203,204], Takahiro Tabuchi[205], Ken Takahashi[113,206], Yonatal Mesfin Tefera[207,208], Mohamad-Hani Temsah[122,209], Marcos Roberto Tovani-Palone[210], Bach Xuan Tran[211], Khanh Bao Tran[212,213], Irfan Ullah[214,215], Pascual R Valdez[216,217], Tommi Juhani Vasankari[218], Francesco S Violante[219,220], Vasily Vlassov[221], Giang Thu Vu[78], Yasir Waheed[222], Yuan-Pang Wang[223], Alex Yeshaneh[224], Biruck Desalegn Yirsaw[225], Engida Yisma[226], Naohiro Yonemoto[227], Mahmoud Yousefifard[228], Chuanhua Yu[229,230], Vesna Zadnik[231], Mohammad Zamani[232], Hamed Zandian[233], Sanjay Zodpey[234], and Stephen S Lim[45,75].

Affilations

1 Sydney School of Public Health, University of Sydney, Sydney, NSW, Australia. .
2 Department of Epidemiology and Biostatistics, Imperial College London, London, UK. .
3 Rollins School of Public Health, Emory University, Atlanta, GA, USA. .
4 School of Health Sciences, University of Manchester, Manchester, UK. .
5 Section of Evidence Synthesis and Classification, International Agency for Research on Cancer, Lyon, France. .
6 Department of Medical Laboratory Science, Haramaya University, Harar, Ethiopia. .
7 Department of Oncology, University of Calgary, Calgary, AB, Canada. .
8 Department of Oncology, Ain Shams University, Cairo, Egypt,. .
9 Department of Preventive Medicine, Dongguk University, Gyeongju, South Korea. .
10 Department of Community Medicine, Kathmandu University, Devdaha, Nepal,. .
11 Endocrinology and Metabolism Research Center, Tehran University of Medical Sciences, Tehran, Iran. .
12 Evidence Based Practice Center, Mayo Clinic Foundation for Medical Education and Research, Rochester, MN, USA. .
13 Internal Medicine Department, Washington University in St. Louis, St Louis, MO, USA. .
14 Clinical Epidemiology Center, VA Saint Louis Health Care System, Department of Veterans Affairs, St Louis, MO, United States,. .
15 Social Determinants of Health Research Center, Rafsanjan University of Medical Sciences, Rafsanjan, Iran. .
16 School of Health Sciences, Birmingham City University, Birmingham, UK. .
17 Department of Community Health Sciences, University of Manitoba, Winnipeg, MB, Canada. .
18 The Judith Lumley Centre, La Trobe University, Melbourne, VIC, Australia. .
19 General Office for Research and Technological Transfer, Peruvian National Institute of Health, Lima, Peru,. .
20 Public Health Risk Sciences Division, Public Health Agency of Canada, Toronto, ON, Canada. .
21 Department of Nutritional Sciences, University of Toronto, Toronto, ON, Canada,. .
22 Social Determinants of Health Research Center, Lorestan University of Medical Sciences, Khorramabad, Iran. .
23 Hepatitis Research Center, Lorestan University of Medical Sciences, Khorramabad, Iran. .
24 Emergency Hospital of Bucharest, Carol Davila University of Medicine and Pharmacy, Bucharest, Romania. .
25 Department of General Surgery, Carol Davila University of Medicine and Pharmacy, Bucharest, Romania. .
26 Department of Statistical and Computational Genomics, National Institute of Biomedical Genomics, Kalyani, India. .
27 Department of Statistics, University of Calcutta, West Bengal, India. .
28 Social Determinants of Health Research Center, Babol University of Medical Sciences, Babol, Iran. .
29 Department of Global Public Health and Primary Care, University of Bergen, Bergen, Norway. .
30 Cancer Registry of Norway, Oslo, Norway,. .
31 School of Population and Public Health, University of British Columbia, Vancouver, BC, Canada. .
32 Al Shifa School of Public Health, Al Shifa Trust Eye Hospital, Rawalpindi, Pakistan,. .
33 Department of Medical Epidemiology and Biostatistics, Karolinska Institutet, Stockholm, Sweden. .
34 Applied Molecular Biosciences Unit, University of Porto, Porto, Portugal. .
35 Institute of Public Health, University of Porto, Porto, Portugal,. .

36 Colombian National Health Observatory, National Institute of Health, Bogota, Colombia. .
37 Epidemiology and Public Health Evaluation Group, National University of Colombia, Bogota, Colombia,. .
38 Mary MacKillop Institute for Health Research, Australian Catholic University, Melbourne, VIC, Australia. .
39 School of Public Health, University of Hong Kong, Hong Kong, China,. .
40 Department of Surgical Oncology, Tata Memorial Hospital, Mumbai, India. .
41 Institute of Industrial Ecological Science, University of Occupational and Environmental Health, Kitakyushu, Japan. .
42 Board of Directors, Food Ingredient and Health Research Institute, Naalehu, HI, USA. .
43 Division of Plastic and Reconstructive Surgery, University of Washington, Seattle, WA, USA. .
44 Public Health Foundation of India, Gurugram, India. .
45 Institute for Health Metrics and Evaluation, University of Washington, Seattle, WA, USA. .
46 Institute for Global Health Innovations, Duy Tan University, Hanoi, Vietnam. .
47 Toxoplasmosis Research Center, Mazandaran University of Medical Sciences, Sari, Iran. .
48 School of Nutrition, Food Science and Technology, Hawassa University, Hawassa, Ethiopia. .
49 Department of Community Medicine, University of Peradeniya, Peradeniya, Sri Lanka. .
50 Deputy of Research and Technology, Ministry of Health and Medical Education, Tehran, Iran. .
51 Department of Clinical Epidemiology and Biostatistics, University of Newcastle, Newcastle, NSW, Australia. .
52 Department of Public Health Sciences, Karolinska Institutet, Stockholm, Sweden. .
53 College of Medicine, Imam Muhammad Ibn Saud Islamic University, Riyadh, Saudi Arabia. .
54 REQUIMTE/LAQV, University of Porto, Porto, Portugal. .
55 Department of Psychiatry, Kaiser Permanente, Fontana, CA, USA. .
56 Department of Health Sciences, A.T. Still University, Mesa, AZ, United States,. .
57 Department of Public Health Medicine, Bielefeld University, Bielefeld, Germany. .
58 Gene Expression & Regulation Program, Cancer Institute (W.I.A), Philadelphia, PA, USA. .
59 Department of Dermatology, Kobe University, Kobe, Japan,. .
60 Department of Environmental Health Science, Mario Negri Institute for Pharmacological Research, Milan, Italy. .
61 School of Pharmacy, Mekelle University, Mekelle, Ethiopia. .
62 Department of Biostatistics, Mekelle University, Mekelle, Ethiopia. .
63 School of Medicine, Boston University, Boston, MA, USA. .
64 School of Dentistry, State University of Montes Claros, Montes Claros, Brazil. .
65 March of Dimes, Arlington, VA, USA. .
66 School of Public Health, West Virginia University, Morgantown, WV, USA. .
67 Department of Radiology, Johns Hopkins University, Baltimore, MD, USA. .
68 School of Medicine, Tehran University of Medical Sciences, Tehran, Iran,. .
69 Department of Pharmacology, Tehran University of Medical Sciences, Tehran, Iran. .
70 Obesity Research Center, Research Institute for Endocrine Sciences, Shahid Beheshti University of Medical Sciences, Tehran, Iran. .
71 Department of Family and Community Medicine, Arabian Gulf University, Manama, Bahrain. .
72 School of Health and Environmental Studies, Hamdan Bin Mohammed Smart University, Dubai, United Arab Emirates. .
73 School of Nursing and Midwifery, Tabriz University of Medical Sciences, Tabriz, Iran. .
74 Independent Consultant, Tabriz, Iran. .
75 Department of Health Metrics Sciences, School of Medicine, University of Washington, Seattle, WA, USA. .
76 Department of Neurology, Cairo University, Cairo, Egypt. .
77 Department of Public Health, Mizan-Tepi University, Teppi, Ethiopia. .
78 Center of Excellence in Behavioral Medicine, Nguyen Tat Thanh University, Ho Chi Minh, Vietnam. .

79 Department of Pediatrics, Dell Medical School, University of Texas Austin, Austin, TX, USA. .
80 Department of Epidemiology and Population Health, Albert Einstein College of Medicine, Bronx, NY, USA. .
81 Department of Epidemiology and Biostatistics, Tehran University of Medical Sciences, Tehran, Iran. .
82 Department of Internal Medicine, Bucharest Emergency Hospital, Bucharest, Romania,. .
83 Department of Legal Medicine and Bioethics, Carol Davila University of Medicine and Pharmacy, Bucharest, Romania. .
84 Department of Clinical Legal Medicine, National Institute of Legal Medicine Mina Minovici, Bucharest, Romania,. .
85 Research Institute for Endocrine Sciences, Shahid Beheshti University of Medical Sciences, Tehran, Iran. .
86 Non-communicable Diseases Research Center, Tehran University of Medical Sciences, Tehran, Iran. .
87 Institute for Physical Activity and Nutrition, Deakin University, Burwood, VIC, Australia. .
88 Sydney Medical School, University of Sydney, Sydney, NSW, Australia. .
89 Department of Global Health, Economics and Policy, Faculty of Medical Sciences, University of Kragujevac, Kragujevac, Serbia. .
90 Department of Community Medicine, Banaras Hindu University, Varanasi, India. .
91 Institute of Family Medicine and Public Health, University of Tartu, Tartu, Estonia. .
92 Department of Nutrition and Dietetics, Mekelle University, Mekelle, Ethiopia. .
93 Immunogenetics Research Center, Mazandaran University of Medical Sciences, Sari, Iran. .
94 Department of Neurology, Mazandaran University of Medical Sciences, Sari, Iran. .
95 Hematology-Oncology and Stem Cell Transplantation Research Center, Tehran University of Medical Sciences, Tehran, Iran. .
96 Hematologic Malignancies Research Center, Tehran University of Medical Sciences, Tehran, Iran. .
97 School of Nursing and Midwifery, Hawassa University, Hawassa, Ethiopia. .
98 Department of Public Health and Community Medicine, Jordan University of Science and Technology, Ramtha, Jordan. .
99 Social Determinants of Health Research Center, Ahvaz Jundishapur University of Medical Sciences, Ahvaz, Iran. .
100 Epidemiology and Biostatistics Department, Health Services Academy, Islamabad, Pakistan. .
101 International Otorhinolaryngology Research Association (IORA), Universal Scientific Education and Research Network (USERN), Tehran, Iran. .
102 Department of Nutrition and Health Science, Ball State University, Muncie, IN, USA. .
103 Clinical Epidemiology Unit, Lund University, Lund, Sweden. .
104 School of Medicine, Xiamen University Malaysia, Sepang, Malaysia. .
105 Pengiran Anak Puteri Rashidah Sa'adatul Bolkiah Institute of Health Sciences , Universiti Brunei Darussalam, Bandar Seri Begawan, Brunei. .
106 Saw Swee Hock School Of Public Health , National University of Singapore, Singapore. .
107 Independent Consultant, Jakarta, Indonesia. .
108 CIBERSAM, San Juan de Dios Sanitary Park, Barcelona, Spain. .
109 Catalan Institution for Research and Advanced Studies (ICREA), Barcelona, Spain,. .
110 Department of Internal Medicine, Post Graduate Institute of Medical Education and Research, Chandigarh, India. .
111 Department of Community and Family Medicine, Academy of Medical Science, Baghdad, Iraq. .
112 Department of Public Health, Maragheh University of Medical Sciences, Maragheh, Iran. .
113 Asbestos Diseases Research Institute, University of Sydney, Sydney, NSW, Australia. .
114 School of Public Health, University of Haifa, Haifa, Israel. .
115 Department of Public Health, Trnava University, Trnava, Slovakia. .

116 Digestive Diseases Research Institute, Tehran University of Medical Sciences, Tehran, Iran. .
117 Non-communicable Diseases Research Center, Shiraz University of Medical Sciences, Shiraz, Iran,. .
118 Department of Maternal and Child Nursing and Public Health, Universidade Federal de Minas Gerais, Belo Horizonte, Brazil. .
119 Department of Medical Laboratory Science, Bahir Dar University, Bahir Dar, Ethiopia. .
120 Institute of Public Health, University of Gondar, Ethiopia. .
121 Research Department, Prince Mohammed Bin Abdulaziz Hospital, Ministry of Health, Riyadh, Saudi Arabia. .
122 College of Medicine, Alfaisal University, Riyadh, Saudi Arabia,. .
123 Peru Country Office, United Nations Population Fund (UNFPA), Lima, Peru. .
124 School of Pharmacy, Haramaya University, Harar, Ethiopia. .
125 Department of Pharmacy, Wollo University, Dessie, Ethiopia. .
126 Breast Surgery Unit, Helsinki University Hospital, Helsinki, Finland. .
127 Clinical Microbiology and Parasitology Unit, Dr. Zora Profozic Polyclinic, Zagreb, Croatia. .
128 University Centre Varazdin, University North, Varazdin, Croatia. .
129 Center for Innovation in Medical Education, Pomeranian Medical University, Szczecin, Poland. .
130 Department of Hypertension, Pomeranian Medical University, Szczecin, Poland. .
131 Faculty of General Medicine, Kyrgyz State Medical Academy, Bishkek, Kyrgyzstan. .
132 Department of Atherosclerosis and Coronary Heart Disease, National Center of Cardiology and Internal Disease, Bishkek, Kyrgyzstan. .
133 Institute of Public Health, Heidelberg University, Heidelberg, Germany. .
134 Institute of Addiction Research (ISFF), Frankfurt University of Applied Sciences, Frankfurt, Germany. .
135 Health Systems and Policy Research Unit, Ahmadu Bello University, Zaria, Nigeria. .
136 Heidelberg Institute of Global Health, Heidelberg University, Heidelberg, Germany,. .
137 Iran National Institute of Health Research, Tehran University of Medical Sciences, Tehran, Iran. .
138 Department of Public Health Medicine, University of KwaZulu-Natal, Durban, South Africa. .
139 Health Sciences Research Center, Mazandaran University of Medical Sciences, Sari, Iran. .
140 Social Determinants of Health Research Center, Kurdistan University of Medical Sciences, Sanandaj, Iran. .
141 Department of Epidemiology and Biostatistics, Kurdistan University of Medical Sciences, Sanandaj, Iran,. .
142 International Laboratory for Air Quality and Health, Queensland University of Technology, Brisbane, QLD, Australia. .
143 Department of Surgery, University of Washington, Seattle, WA, USA. .
144 Department of Health Management and Economics, Tehran University of Medical Sciences, Tehran, Iran. .
145 Department of Pediatric Medicine, Nishtar Medical University, Multan, Pakistan. .
146 Department of Pediatrics, Institute of Mother & Child Care, Multan, Pakistan,. .
147 General Surgery Department, Carol Davila University of Medicine and Pharmacy, Bucharest, Romania. .
148 Anatomy and Embryology Department, Carol Davila University of Medicine and Pharmacy, Bucharest, Romania. .
149 Department of Cardiology, Cardio-Aid, Bucharest, Romania,. .
150 Public Health Science Department, State University of Semarang, Kota Semarang, Indonesia. .
151 Graduate Institute of Biomedical Informatics, Taipei Medical University, Taipei City, Taiwan,. .
152 Centre of Cardiovascular Research and Education in Therapeutics, Monash University, Melbourne, VIC, Australia. .

153 Independent Consultant, Accra, Ghana. .
154 Translational Health Research Institute, Western Sydney University, Penrith, NSW, Australia. .
155 Department of Psychiatry and Behavioural Neurosciences, McMaster University, Hamilton, ON, Canada. .
156 Department of Psychiatry, University of Lagos, Lagos, Nigeria,. .
157 Centre for Healthy Start Initiative, Lagos, Nigeria. .
158 Center for Health Systems Research, National Institute of Public Health, Cuernavaca, Mexico. .
159 Department of TB & Respiratory Medicine, Jagadguru Sri Shivarathreeswara University, Mysore, India. .
160 Department of Medical Humanities and Social Medicine, Kosin University, Busan, South Korea. .
161 Cartagena University, Cartagena, Colombia. .
162 Department of Nephrology, Sanjay Gandhi Postgraduate Institute of Medical Sciences, Lucknow, India. .
163 Non-communicable Diseases Research Center, Alborz University of Medical Sciences, Karaj, Iran. .
164 A.T. Still University, Mesa, AZ, USA. .
165 MEDICHEM, Barcelona, Spain. .
166 Department of Epidemiology and Biostatistics, Contech School of Public Health, Lahore, Pakistan. .
167 Contech International Health Consultants, Lahore, Pakistan. .
168 Department of Immunology, Mazandaran University of Medical Sciences, Sari, Iran. .
169 Molecular and Cell Biology Research Center, Mazandaran University of Medical Sciences, Sari, Iran. .
170 Thalassemia and Hemoglobinopathy Research Center, Ahvaz Jundishapur University of Medical Sciences, Ahvaz, Iran. .
171 Clinical Research Development Unit, Golestan Hospital, Ahvaz Jundishapur University of Medical Sciences, Ahvaz, Iran. .
172 Sina Trauma and Surgery Research Center, Tehran University of Medical Sciences, Tehran, Iran. .
173 Department of Health Education and Promotion, Kermanshah University of Medical Sciences, Kermanshah, Iran. .
174 School of Social Sciences and Psychology, Western Sydney University, Penrith, NSW, Australia. .
175 Environmental Determinants of Health Research Center, Kermanshah University of Medical Sciences, Kermanshah, Iran. .
176 Department of Clinical Research, Federal University of Uberlândia, Uberlândia, Brazil. .
177 Department of Cardiology, Tehran University of Medical Sciences, Tehran, Iran. .
178 Medical Department, University of Sharjah, Sharjah, United Arab Emirates. .
179 Emergency Department, Shahid Beheshti University of Medical Sciences, Tehran, Iran. .
180 Department of Epidemiology and Biostatistics, School of Public Health, Tabriz University of Medical Sciences, Tabriz, Iran. .
181 Biotechnology Research Center, Mashhad University of Medical Sciences, Mashhad, Iran. .
182 Neurogenic Inflammation Research Center, Mashhad University of Medical Sciences, Mashhad, Iran. .
183 Multiple Sclerosis Research Center, Tehran University of Medical Sciences, Tehran, Iran. .
184 Department of Entomology, Ain Shams University, Cairo, Egypt. .
185 Department of Surgery, Marshall University, Huntington, WV, USA. .
186 Department of Nutrition and Preventive Medicine, Case Western Reserve University, Cleveland, OH, USA. .
187 Department of Health and Society, Faculty of Medicine, University of Applied and Environmental Sciences, Bogotá, Colombia. .

188 Surgery Department, Hamad Medical Corporation, Doha, Qatar. .
189 Faculty of Health & Social Sciences, Bournemouth University, Bournemouth, UK. .
190 Department of Psychology, University of Alabama at Birmingham, Birmingham, AL, USA. .
191 Environmental Health Associates LLC, Englewood, CO, USA. .
192 School of Public Health, University of Colorado Denver, Denver, CO, USA. .
193 Independent Consultant, Karachi, Pakistan. .
194 University School of Management and Entrepreneurship, Delhi Technological University, New Delhi, India. .
195 Department of Pulmonary Medicine, Fudan University, Shanghai, China. .
196 National Institute of Infectious Diseases, Tokyo, Japan. .
197 Cancer Research Institute, Tehran University of Medical Sciences, Tehran, Iran. .
198 Cancer Biology Research Center, Tehran University of Medical Sciences, Tehran, Iran. .
199 School of Public Health and Preventive Medicine, Monash University, Melbourne, VIC, Australia. .
200 Department of Epidemiology, School of Preventive Oncology, Patna, India. .
201 Social Development and Health Promotion Research Center, Kermanshah University of Medical Sciences, Kermanshah, Iran. .
202 Department of Psychology, Deakin University, Burwood, VIC, Australia. .
203 Department of Medicine, University of Valencia, Valencia, Spain. .
204 Carlos III Health Institute, Biomedical Research Networking Center for Mental Health Network (CiberSAM), Madrid, Spain,. .
205 Cancer Control Center, Osaka International Cancer Institute, Osaka, Japan. .
206 University of Sydney, Syndey, NSW, Australia. .
207 School of Public Health, University of Adelaide, Adelaide, SA, Australia. .
208 Department of Environmental Health, Wollo University, Dessie, Ethiopia,. .
209 Department of Pediatrics, King Saud University, Riyadh, Saudi Arabia. .
210 Department of Pathology and Legal Medicine, University of São Paulo, Ribeirão Preto, Brazil. .
211 Department of Health Economics, Hanoi Medical University, Hanoi, Vietnam. .
212 Department of Molecular Medicine and Pathology, Auckland Cancer Society Research Centre, University of Auckland, Auckland, New Zealand. .
213 Maurice Wilkins Centre for Biodiscovery, Auckland, New Zealand. .
214 Gomal Center of Biochemistry and Biotechnology, Gomal University, Dera Ismail Khan, Pakistan. .
215 TB Culture Laboratory, Mufti Mehmood Memorial Teaching Hospital, Dera Ismail Khan, Pakistan. .
216 Argentine Society of Medicine, Buenos Aires, Argentina. .
217 Velez Sarsfield Hospital, Buenos Aires, Argentina,. .
218 UKK Institute, Tampere, Finland. .
219 Department of Medical and Surgical Sciences, University of Bologna, Bologna, Italy. .
220 Occupational Health Unit, Sant'Orsola Malpighi Hospital, Bologna, Italy. .
221 Department of Health Care Administration and Economy, National Research University Higher School of Economics, Moscow, Russia. .
222 Foundation University Medical College, Foundation University, Rawalpindi, Pakistan. .
223 Department of Psychiatry, University of São Paulo, São Paulo, Brazil. .
224 Department of Midwifery, Wolkite University, Wolkite, Ethiopia. .
225 University of South Australia, Adelaide, NSW, Australia. .
226 School of Allied Health Sciences, Addis Ababa University, Addis Ababa, Ethiopia. .
227 Department of Psychopharmacology, National Center of Neurology and Psychiatry, Tokyo, Japan. .
228 Physiology Research Center, Iran University of Medical Sciences, Tehran, Iran. .
229 Department of Epidemiology and Biostatistics, Wuhan University, Wuhan, China. .

230 Global Health Institute, Wuhan University, Wuhan, China,. .
231 Epidemiology and Cancer Registry Sector, Institute of Oncology Ljubljana, Ljubljana, Slovenia. .
232 Student Research Committee, Babol University of Medical Sciences, Babol, Iran. .
233 Social Determinants of Health Research Center, Ardabil University of Medical Science, Ardabil, Iran. .
234 Indian Institute of Public Health, Public Health Foundation of India, Gurugram, India. .
235 ISGlobal, Barcelona, Spain
